# Structural variant analysis of a cancer reference cell line sample using multiple sequencing technologies

**DOI:** 10.1186/s13059-022-02816-6

**Published:** 2022-12-13

**Authors:** Keyur Talsania, Tsai-wei Shen, Xiongfong Chen, Erich Jaeger, Zhipan Li, Zhong Chen, Wanqiu Chen, Bao Tran, Rebecca Kusko, Limin Wang, Andy Wing Chun Pang, Zhaowei Yang, Sulbha Choudhari, Michael Colgan, Li Tai Fang, Andrew Carroll, Jyoti Shetty, Yuliya Kriga, Oksana German, Tatyana Smirnova, Tiantain Liu, Jing Li, Ben Kellman, Karl Hong, Alex R. Hastie, Aparna Natarajan, Ali Moshrefi, Anastasiya Granat, Tiffany Truong, Robin Bombardi, Veronnica Mankinen, Daoud Meerzaman, Christopher E. Mason, Jack Collins, Eric Stahlberg, Chunlin Xiao, Charles Wang, Wenming Xiao, Yongmei Zhao

**Affiliations:** 1 Sequencing Facility Bioinformatics Group, Advanced Biomedical and Computational Science, Frederick National Laboratory for Cancer Research, Frederick, MD USA; 2 Bioinformatics and Computational Science Directorate, Frederick National Laboratory for Cancer Research, Frederick, MD USA; 3 Illumina Inc, Foster City, CA USA; 4 Sentieon Inc, Mountain View, CA USA; 5 Center for Genomics, Loma Linda University School of Medicine, Loma Linda, CA USA; 6 Sequencing Facility, Cancer Research Technology Program, Frederick National Laboratory for Cancer Research, Frederick, MD USA; 7 Immuneering Corp, Cambridge, MA USA; 8 Laboratory of Human Carcinogenesis, Center for Cancer Research, National Cancer Institute, Bethesda, MD USA; 9 Bionano Genomics, San Diego, CA92121 USA; 10 Department of Allergy and Clinical Immunology, State Key Laboratory of Respiratory Disease, Guangzhou Institute of Respiratory Health, the First Affiliated Hospital of Guangzhou Medical University, Guangzhou, Guangdong China; 11 Center for Drug Evaluation and Research, FDA, Silver Spring, MD USA; 12 Bioinformatics Research & Early Development, Roche Sequencing Solutions Inc, 1301 Shoreway Road, Belmont, CA 94002 USA; 13 DNAnexus, Mountain View, CA USA; 14 Dovetail Genomics, Scotts Valley, CA USA; 15 Computational Genomics and Bioinformatics Branch, Center for Biomedical Informatics and Information Technology (CBIIT), National Cancer Institute, Rockville, MD USA; 16 Department of Physiology and Biophysics, Weill Cornell Medicine, New York, NY USA; 17 National Center for Biotechnology Information, National Library of Medicine, National Institutes of Health, Bethesda, MD USA

**Keywords:** Structural variation, Reference call set, Cancer, Multiple platforms, Structural variant calling algorithm, Next-generation sequencing technology

## Abstract

**Background:**

The cancer genome is commonly altered with thousands of structural rearrangements including insertions, deletions, translocation, inversions, duplications, and copy number variations. Thus, structural variant (SV) characterization plays a paramount role in cancer target identification, oncology diagnostics, and personalized medicine. As part of the SEQC2 Consortium effort, the present study established and evaluated a consensus SV call set using a breast cancer reference cell line and matched normal control derived from the same donor, which were used in our companion benchmarking studies as reference samples.

**Results:**

We systematically investigated somatic SVs in the reference cancer cell line by comparing to a matched normal cell line using multiple NGS platforms including Illumina short-read, 10X Genomics linked reads, PacBio long reads, Oxford Nanopore long reads, and high-throughput chromosome conformation capture (Hi-C). We established a consensus SV call set of a total of 1788 SVs including 717 deletions, 230 duplications, 551 insertions, 133 inversions, 146 translocations, and 11 breakends for the reference cancer cell line. To independently evaluate and cross-validate the accuracy of our consensus SV call set, we used orthogonal methods including PCR-based validation, Affymetrix arrays, Bionano optical mapping, and identification of fusion genes detected from RNA-seq. We evaluated the strengths and weaknesses of each NGS technology for SV determination, and our findings provide an actionable guide to improve cancer genome SV detection sensitivity and accuracy.

**Conclusions:**

A high-confidence consensus SV call set was established for the reference cancer cell line. A large subset of the variants identified was validated by multiple orthogonal methods.

**Supplementary Information:**

The online version contains supplementary material available at 10.1186/s13059-022-02816-6.

## Background

Genomic instability is a key hallmark of cancer [1], underpinned by translocations, large insertions/deletions, and inversions of >50bp in size, which together are referred to as SVs [2]. An SV in a cell can nefariously activate a cancerous gene [3] or block a tumor regulatory gene [4]. In order to accurately detect cancer, monitor relapse, develop a precision medicine strategy, or enable drug discovery and development, accurate detection of somatic SVs is a driving imperative.

Out of all somatic variants that can be cancer driver events, SVs can be the most challenging to detect using NGS due to the repetitive structure of the human genome [5, 6]. Current clinical testing utilizes G-band karyotyping, PCR, and fluorescence in situ hybridization (FISH), all of which are low-throughput technologies that require prior knowledge of the SV’s genomic location [7–9]. The advent of microarrays has increased throughput, but microarrays lack the base pair resolution to pinpoint the precise start and stop locations of rearrangements [10].

Next-generation sequencing (NGS) technologies show great promise for future clinical utility in somatic SV detection due to their single-nucleotide level resolution and high throughput [11, 12]. Current short-read NGS technologies are more accurate at base calling but struggle to detect SVs in highly repetitive or low-complexity regions [13, 14]. Long-read technologies can easily span break points, but have a high sequencing error rate from raw data (~10–15%) although the latest chemistry from long-read technologies such as PacBio HiFi reads can achieve greater accuracy (>99.9%) [15]. Therefore, integrating data from multiple technologies is necessary for the most accurate SV detection. We applied different bioinformatic methods that incorporate both short-read and long-read technologies to create a reference call set for somatic SVs.

Previous studies have integrated Hi-C and WGS to resolve and phase SVs in cancer samples [16]. Recent in silico work added SVs using BAMSurgeon [17] and then benchmarked and analyzed 204 versions of SV calling bioinformatics pipelines [18]. Others have studied distinctive structural features including genome rearrangements using discordantly mapping paired-end reads from short-read WGS data of >500 breast cancer samples [12]. Focusing on a breast cancer cell line, a previous study found over 20,000 structural variants by leveraging long-read technology combined with RNA-seq [19, 20]. However, key mutation characteristics in this cancer cell line, such as single-nucleotide variants (SNVs) and small indels, have not been well-studied. Taken together, previous studies showed great promise for SV detection approaches but also showcased the need for targeted investigation to elucidate the deep complexity of the breast cancer genome.

A pair of tumor/normal matched cell lines was studied and well-characterized in other companion studies by the SEQC2 Consortium as reference samples. A reference call set for SNVs/indels was provided for benchmarking the performances of WGS, WES, and single-cell RNA-seq across different technologies/platforms [21–23]. As a companion study, here we combined short-read, long-read, as well as long-range mapping data types to better resolve SVs and additionally compare technologies and bioinformatics pipelines for accurate SV calling in this well-described pair of tumor-normal cell lines [22]. We produced a consensus SV call set for the reference cancer cell line, which was validated using four orthogonal technologies. This SV call set will be a valuable resource to the community for benchmarking and/or developing of SV detection methods and algorithms.

Given the complexity of sequencing, analyzing, and integrating SV calling, more studies are needed to characterize and understand best practices to enhance the effectiveness of cancer SV detection for the research community and in the clinic.

## Results

### Overall study design

**Fig. 1:**
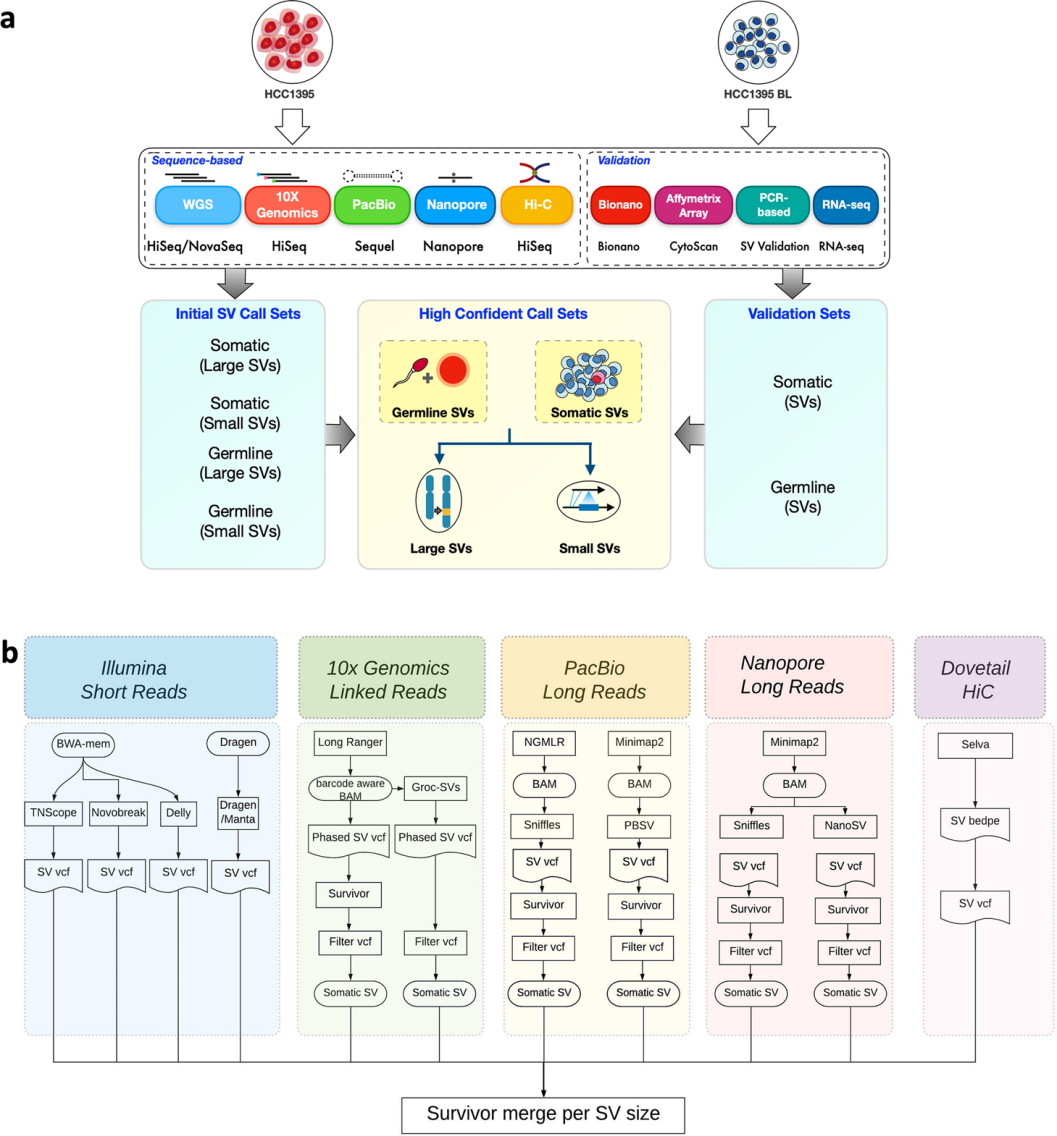
Study design and bioinformatics workflow for SV detection and integration. **a** Schematic overview of the study design. Two well-characterized reference cell lines (HCC1395 and HCC1395BL) were used to generate whole-genome sequencing data across five platforms (Illumina short reads, 10X Genomics linked reads, PacBio long reads, Oxford Nanopore long reads, and Dovetail Hi-C proximity ligation). Initial SV call sets were identified from each platform and combined together to identify high-confidence call sets. The SVs from high-confidence call sets were selected for PCR-based validation for deletion, insertion, intra-chromosomal inversion and inter-chromosomal translocation; copy number changes were validated by Affymetrix array. Large SVs (≥20kb) were validated using Bionano optical mapping. RNA-seq was used to validate the fusion gene and translocation events. **b** Schematic overview of the bioinformatics analysis workflow. Each platform’s data was processed by the aligner and SV caller specific to that platform. The tumor-only or somatic SV calls were selected by Survivor. The final call sets from each platform were integrated together using the Survivor software tool based a window-size approach and SV types

To establish the consensus SV call set and evaluate the factors that impact SV detection accuracy in cancer, we compared different NGS technologies, library protocols, and bioinformatics pipelines for SV calling. We chose a breast cancer cell line with a matched normal cell line (tumor HCC1395 and normal HCC1395BL) previously used by our consortium [22] for SNV profiling. We generated sequencing data from multiple technologies including Illumina short-reads, 10X Genomics linked reads, PacBio single-molecule long reads, Oxford nanopore long reads, and high-throughput chromosome conformation capture (Hi-C) (Fig. 1a and Table 1). The NGS libraries include the following: 21 pairs of the tumor and normal samples which were prepared with Illumina’s TruSeq DNA PCR-free library prep kit and sequenced at ~50X for each library; 21 pairs of mixed tumor and normal samples prepared using Illumina’s TruSeq DNA PCR-Free library preparation were sequenced at ~110X for each library to study the impact of tumor purity on the SV profile; 11 pairs of the tumor and normal samples were prepared with 10X Chromium Genome V2 kit and were sequenced at 80X for each library; one pair of the PacBio Sequel 20kb libraries were sequenced at 39X and 44X for HCC1395 and HCC1395BL, respectively; Oxford Nanopore MinION sequencing of HCC1395 at 12X and HCC1395BL at 19X ; three replicates of all libraries for each cell line were made from Dovetail Hi-C library preparation and were sequenced at 37X and 34X for HCC1395 and HCC1395BL, respectively (Additional file 1: Table S1). Across all of our efforts, this study obtained over 6600X genome coverage for HCC1395 and HCC1395BL. We built an in-house pipeline that integrated results from five NGS platforms. The pipeline included TNscope, novoBreak, Delly, and Manta for calling SVs from Illumina short reads; Long Ranger and GrocSVs for calling SVs from 10X Genomics linked reads, PBSV and Sniffles for calling SVs from PacBio long reads, Sniffles and NanoSV for calling SVs from Nanopore long reads, and Selva for SV discovery from Dovetail Hi-C proximity ligation data (Fig. 1b). As a result, we generated a total of 82 structural variant call sets which included small SVs (defined as 50bp ≤ DNA SV size and ≤30kb) and large SVs (defined as DNA variants > 30kb) (Additional file 2: Table S2). Previous studies of this breast cancer cell line have revealed many somatic structural and ploidy changes [22, 24]. In this manuscript, we focused on somatic SVs, comparing the tumor HCC1395 cell line to the matched HCC1395BL cell line. After integrating the results from each platform (see “Methods”), we identified initial SV call sets (Additional file 3: Table S3). We compiled a list of high-quality consensus SVs based on consensus scores across platforms and analytical methods (Additional file 4: Table S4). In order to obtain validation rates for the SVs in the consensus call sets, we selected a subset of SVs from our high-confidence consensus call set and initial call set including SVs called by multiple technologies or platform-specific call sets for validation using three different methods including Bionano, PCR-based analysis, and Affymetrix array for validation (Additional file 5, 6, 7 and 8: Table S5-8). In addition, we identified fusion transcripts using RNA-seq to validate a subset of SVs (Additional file 9: Table S9). This demonstrated the unique strength of integrative analysis from multi-platform as well as multi-analytical methods. We documented a great variety of notable mutations including complex rearrangements and gene fusion events. Moreover, the bioinformatics analysis methods and pipelines we present here could easily be adapted for future studies, especially for multi-platform data integration for cancer genome SV profiling.

**Table 1:** Sequencing summary from multiple NGS technologies. Whole-genome and transcriptome sequencing data from multiple NGS platform for structural variants (SVs) and fusion gene detection in HCC1395 and HCC1395BL reference samples

NGS Technologies	Materials	Library protocols	Average library insert sizes	Total number of reads (genome coverage)	
				HCC1395	HCC1395BL
**Illumina WGS on HiSeq/NovaSeq/X10**	**Fresh DNA**	**Illumina TruSeq DNA PCR Free 1μg Input Library Prep**	**400bp**	**35 billion (1,148X)**	**34 billion (1,119X)**
	**Fresh DNA with mixture of tumor and normal cells for purity study**	**Illumina TruSeq DNA PCR Free 1μg Input Library Prep**	**400bp**	**64 billion (2348X)**	
**10X Genomics WGS**	**Fresh DNA**	**10X Genomics Chromium Genome V2 kit**	**65kb**	**23 billion (974X)**	**21 billion (881X)**
**PacBio WGS on Sequel**	**Fresh DNA**	**PacBio Sequel 10 kb Library Prep with Sequel Chemistry Kits v 2.1**	**10kb**	**116 million (39X)**	**132 million (44X)**
**Hi-C WGS on NextSeq**	**Fresh DNA**	**Dovetail SELVA Library Prep Kit**	**NA**	**200 million (34X)**	**200 million (34X)**
**Oxford Nanopore**	**Fresh DNA**	**Oxford Nanopore SQK-LSK109 ligation sequencing kit**	**9kb**	**8 million (12X)**	**20 million (19X)**
**Illumina RNA-seq on NextSeq**	**Fresh RNA**	**NuGEN Ovation RNA-seq Library Prep**	**300bp**	**188 million**	**192 million**

### Establishing consensus somatic SV call set

The main objective of this study is to discover the major classes of SVs such as deletions, insertions, duplications, inversions, and translocations as well as other complex SV events in the reference cancer cell line. In order to increase detection sensitivity to avoid limitations of individual software caller algorithms, we first analyzed each data set for each sample replicate with platform-specific analytical software tools to make 82 raw call sets (Fig. 1b, Additional file 2: Table S2). Next, for integration of SVs from multiple replicates and software tools, we used the Survivor algorithm [25]. We applied different window sizes based on SV sizes and SV types to merge overlapping SVs with breakpoints within the specific window sizes together. For small SVs ranging from 50bp to 30kb, we used window sizes of 50 to 100bp, 100 to 500bp, 500 to 1kb, and deletions greater than 1kb but smaller than 30kb. For SVs larger than 30kb, we used a maximum window size of 10kb between the breakpoints to merge overlapping SVs. SV events such as insertions, deletions, intra-chromosomal inversions, inter-chromosomal translocations, and complex breakpoint events were called in a tool-specific manner (Fig. 1b). Depending on software algorithms, one algorithm may only identify deletion and insertion events, while other algorithms may be able to identify additional SV types. To call tumor-specific SVs, the SVs present exclusively in the tumor sample but not in the normal sample were identified as somatic SVs for each matched pair (“Methods”).

For the Illumina short-read data set, we used BWA-MEM [26] to align reads. Across all replicates, above 99% of Illumina short paired end reads (410 bp fragment length) were aligned to the reference genome. Four variant callers including TNscope, novoBreak, Delly, and Manta (Illumina DRAGEN pipeline) [27] were used for calling the SVs from the same data set. The Survivor algorithm was then used to integrate the SVs from 44 call sets for Illumina short reads and obtain consensus call sets from the four different short-read variant callers. A total of 46,284 somatic SVs were called from HCC1395 and 168,401 germline SVs were called from HCC1395BL. We compared both aligner effect and SV caller effect on the SV detection consistency. The alignment BAM files generated by BWA-MEM and NovaAlign were used as input for TNscope for 24 pairs of Illumina WGS data set with a maximum 150-bp window size for breakpoints. We observed high concordance calls among the two aligners’ results with concordance between 0.67 and 0.91 (Additional file 10: Fig. S1a). For PacBio Sequel sequencing data, the average PacBio read length was about 8kb. We used both PBSV and Sniffles for structural variant calling and Survivor for consensus calling from the two long-read variant callers. A total of 9574 somatic SVs and 27,069 germline SVs were called from HCC1395 and HCC1395BL, respectively. For 10X Genomics linked read data, the average molecule length was 65kb. 10X Genomics’ Long Ranger software was used to call large SVs including insertions, deletions, and translocation events with lengths of 30kb and above. For small SVs, only deletions between 50bp and 30kb are called. We observed very low concordance (0.09–0.13) of Long Ranger SV small deletion calls between 11 pairs of replicates for the 10X Genomics linked read data sets (Additional file 10: Fig. S1b). However, the concordance rates among 11 pairs of replicates for the10X Genomics linked read data set called by Long Ranger and GrocSVs for large SVs (30kb and above) are higher (0.58–0.74) (Additional file 10: Fig. S1c-d). Further analysis showed the lower concordance rates among the Long Ranger SVs were due to a large number of private calls for the same one replicate. Specifically, most SVs were small deletions (50bp–20kb) called in single sample, which were not detected by PacBio platform or called in other 10X Genomics replicate datasets (Additional file 10: Fig. S2). Due to the low concordance rate among 10X Genomics Long Ranger small deletion call sets, we excluded deletions less than 20kb in Long Ranger call sets for further downstream analysis. The consensus calls from Long Ranger and GrocSVs included 16,886 somatic variants and 37,059 germline variants from HCC1395 and HCC1395BL respectively. For Nanopore long reads, a total of 21,245 and 63,061 variants were found in HCC1395 and HCC1395BL, respectively. For the Dovetail Hi-C libraries, there were a total of 137 large SVs from HCC1395 and 1 SV called from HCC1395BL.

**Fig. 2:**
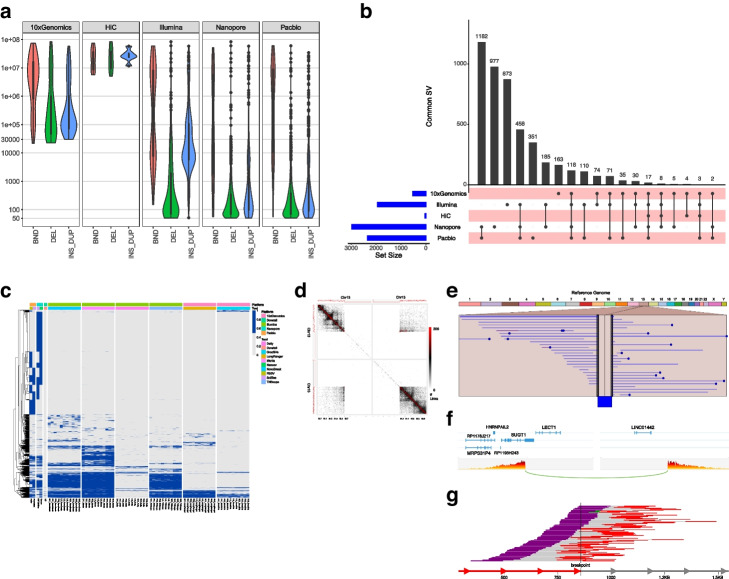
SV detection comparison across different NGS technologies and Software Tools in HCC1395 cancer cell Line. **a** Violin plot shows the SV detection size ranges for all of SVs called by each NGS platform. The *y*-axis denotes the SV size in bp, the *x*-axis denotes the SV types detected by each platform. **b** Comparison of SVs called by different NGS platform among 10X Chromium linked reads, Illumina short reads, Hi-C, Nanopore, and PacBio call sets. The blue horizontal bars on the left side show the total number of SVs in the specific sequencing platform, black dots on the pink bars denote total SVs called in each sequencing platform. The top black vertical bars display the total concordance calls among the different sequencing platforms. **c** The heatmap denotes the SV frequencies detected by each tool and technology were generated based on SV location on genome, SV type, and SV frequency which was calculated based on the section “Calculating SV calling frequency and select high-confidence call set”. The platforms include 10X Genomics, Dovetail Hi-C, Illumina, Nanopore, and PacBio. Software tools in the plots include Dell, GrocSVs, Hi-C (Selva), Long Ranger, Manta, Nanosv, NovoBreak, PBSV, Sniffles Nanopore, Sniffles PacBio, and TNscope. The heatmap color denotes the SV frequencies detected by each tool and technology, the dendrograms along the side of the heatmap show similarity and variability how the SVs are clustered. **d-e** Cross-platform detection of a deletion between 52 and 60Mb region of the chromosome 13. The deletion event was identified by all the replicates and software tools**. d** Hi-C detection of the deletion event. The outer blackline (outside of the contact matrix) suggests the average read coverage across the entire genome. Red line is raw reads coverage per position. Top left and bottom right part of the contact matrix showing the common contact with 2158 total spanning reads. **e** Deletion from PacBio data using Ribbon software. The data mapped by minimap2 caller and SV event called by PBSV and Sniffles. The deletion is shown in the middle. The dots in the plot suggest the indeletion events. There are a total 29 reads showing deletion from the PacBio data. **f** 10X Genomics detected deletion event: Image is generated using loupe browser. The image is showing the barcode interaction between the two coordinates of the chr13 location suggesting deletion. The slope suggests the total number of shared barcodes between two locations. The data was mapped using lariat (Long Ranger) and events called by Long Ranger (SV) and Groc_SVs. **g** The visualization is generated using SVVIZ from Illumina data. Reads aligning better to the alternate allele than the reference allele will be shown in the set of tracks. Line indicates the break point across 79 reads. The data was mapped using BWA, and SV events were called by Delly, Manta, Novobreak, and TNScope

After integrating SVs from all five platforms and removing those overlapping blacklisted regions of the genome, we also filtered out low-quality SVs in addition to SVs that were not present in at least two replicates. We retained a total of 4662 SVs in the initial combined call set (Fig. 2, Additional file 3: Table S3). Our results covered a wide spectrum of events due to the variable size detection ranges of each platform. The Hi-C platform, for instance, can detect SVs greater than 1Mb in size for balanced, unbalanced, and complex events with no upper size limit; however, it cannot detect smaller SVs. Each software algorithm is also limited by the type of SVs it can detect. Taking technology-specific range limitations into consideration, we calculated the overlap of SVs detected by each platform compared to the 4662 SVs in the initial call set. The overlap rates for Illumina, PacBio, Nanopore, 10X Genomics (large SVs ≥20kb only), and Hi-C (large SVs ≥1 Mb) are 43, 50, 63, 43, and 4%, respectively (Additional file 3: Table S3).

**Fig. 3:**
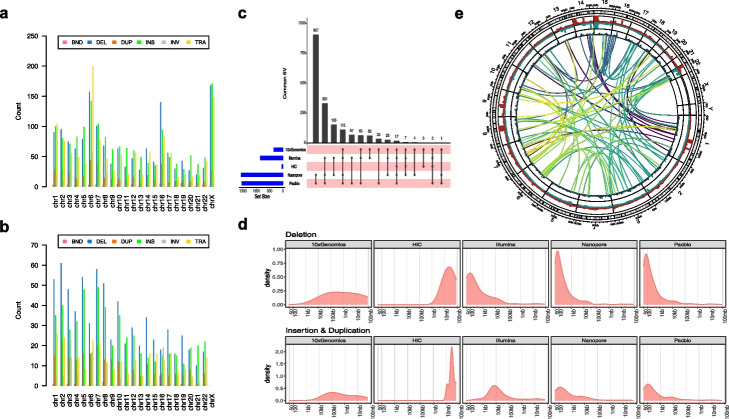
Structural variant initial and high-confidence call set. **a** Bar chart plot displays BNDs, DELs, DUPs, INSs, INVs, and TRA on all chromosomes from initial call set. **b** Bar chart plot displays BNDs, DELs, DUPs, INSs, INVs, and TRA on all chromosomes from high-confidence call set. **c** Upset plot to display the number of SV overlap between the different NGS platforms. The blue horizontal bars on the left side show the total number of SVs in the specific NGS platform, black dots on the pink bars denote total SVs called in each platform. The top black vertical bars display the total concordance calls among the different platforms. **d** Density plot for SV size distributions for Deletion events (top panel) and Duplication events (bottom panel). The *y*-axis denotes log10 scale of number of SVs; the *x*-axis denotes the SV size bin from 50bp to 20Mb. **e** Circos plot visualization of results from the HATCHet + RCK analysis from the matched tumor/normal (HCC1395 and HCC1395BL) WGS analysis. The amplification track (CN > 1, red) and deletion track (CN < 1, blue) show the fraction of the amplified or deleted regions as reported by RCK. The breakpoint bar plot shows the number of novel adjacency (structural variant) breakpoints that start or end within a chromosomal region (max = 128). The center chord diagram shows the start and end points for all inter-chromosomal transversion events (*n* = 122). All structural variants shown are present in the consensus call set. Chromosomal regions for the amplification, deletion, and structural variant breakpoint tracks are binned into 5 megabase windows

Previous work showed that using the Survivor algorithm to form a high-quality consensus call set from at least two different short-read variant callers significantly reduces false-positive rates [25]. In order to build a high-quality consensus call set, we used a similar approach to take the consensus calls from multiple samples and tools and required at least two technologies from two software tools to agree on the same variant based on SV types and SV sizes. In addition, we applied a clustering algorithm to cluster the SVs of the same type and similar size to merge the SVs if they had a reciprocal overlap >50%. We integrated all call sets and calculated consensus scores for each variant based on the frequency of variants detected from orthogonal techniques by multiple software tools. We further removed the SVs on three chromosomal regions with normal loss of heterozygosity (LOH) on chr6p, chr16q, and chrX to obtain a unified SV call set. We performed manual curation of the tumor sample consensus SV call set to obtain the final high-confidence call set consist of 1788 SVs including 717 deletions, 230 duplications, 551 insertions, 133 Inversion and 146 translocations, and 11 multiple breakpoint events (Fig. 3, Table 2, and Additional file 4: Table S4). In our high-confidence consensus call set, 559 (31%) were detected by at least three platforms. Illumina, PacBio, Nanopore, 10X Genomics (large SVs ≥20kb only), and Hi-C (large SVs ≥1 Mb) had each detected 47, 86, 87, 18, and 2% of the final high-confidence call set, respectively. Among all the five platforms, we observed that long-read-based technologies such as PacBio, Nanopore, and 10X Genomics linked reads detected a high number of SVs that are missed by short-read-based technologies (Fig. 2b, c). In addition, the PacBio and Nanopore detected SVs call sets have the highest overlap compared to any other platforms (86% for PacBio and 87% for Nanopore detected SVs in the high-confidence call set). On the other hand, we observed consistency for the SVs discovered by multiple platforms especially for deletions and insertions/duplication events. Figure 2d–g shows one of the deletion events which is located between the 52,000,000 and 60,000,000 region of chromosome 13. The deletion event was identified by all the replicates and software tools for four platforms including PacBio, Illumina, 10X Genomics, and Hi-C technology.

**Table 2:** SVs called from HCC1395 and HCC1395BL cell lines. The somatic SV and germline SV raw calls are the number of SVs counted after Survivor software tool merge of all replicate call sets for each platform. The total merged somatic SV raw calls contain the merged tumor-only calls from tumor-normal paired subtraction analysis. The total merged germline SV calls contain the merged germline SV calls from all replicates from HCC1398BL cell line

NGS Technologies	Initial SVs call set		High-confidence SVs call set					
	HCC1395 (tumor cell line)	HCC1395BL (normal cell line)	HCC1395 (tumor cell line)					
	Somatic SVs (raw calls)	Germline SVs (raw calls)	Deletions	Duplications	Insertions	Inversions	Translocations	BNDs
Illumina WGS	**9155**	**105,732**	**375**	**226**	**4**	**82**	**143**	**10**
10x GenomicsWGS	**16,886**	**37,059**	**42**	**86**	**N/A**	**78**	**119**	**5**
PacBio WGS	**9574**	**27,069**	**643**	**138**	**549**	**94**	**99**	**10**
HiC WGS	**137**	**1**	**8**	**6**	**N/A**	**9**	**5**	**1**
Oxford NanoporeWGS	**21,245**	**63,061**	**664**	**180**	**549**	**65**	**96**	**4**
Total Merged SVs	**46,284**	**168,401**	**717**	**230**	**551**	**133**	**146**	**11**

### Multiple orthogonal methods for SV validation

In order to independently validate tumor cell line SVs and measure the accuracy of our consensus SV call set, we used four orthogonal methods: Bionano optical mapping, Affymetrix array, PCR-based validation, and RNA-seq based fusion gene detection (Fig. 1a, Additional file 5: Table S5). Bionano optical mapping is often used for confirming large SVs discovered by NGS technologies [28]. Bionano molecule lengths typically range from 150kb to a few megabases and thus are substantially longer than the normal sequencing read lengths, enabling detection of SVs of all types between 500bp up to megabases [28]. We detected and confirmed a total of 57 inter-chromosomal translocation events, 60 large deletions and 40 large duplications, and 9 inversion events (Additional file 6: Table S6). For further validation of CNV and other types of structural variants (balanced translocations, tandem duplications, inversions), PCR was used to target the breakpoints associated with a subset of putative SVs. We designed primers based on SV types and validated 29 deletions, 23 duplications, 12 inversion, and 6 translocation events (Additional file 7: Table S7). We used an Affymetrix array (Affymetrix GeneChip Scanner 3000 G7) for measuring copy number gain and loss. There were a total of 40 large CNVs confirmed by microarray including 14 large deletions/copy number losses (>50kb) and 26 large duplications/copy number gains (> 50kb) (Additional file 8: Table S8). We also analyzed the RNA-seq libraries from the same pair of breast cancer cell lines to identify fused gene transcripts (“Methods”, Additional file 9: Table S9). We identified 24 putative fusion gene transcripts, each with at least two split reads that overlap with SVs found in the tumor sample consensus SV call set. Forty-nine large SVs including duplication, translocation, inversion, and deletion events were confirmed by at least two methods, while the rest of the 197 SVs were confirmed by one orthogonal method only (Additional file 10: Fig. S3). Additional file 10: Fig. S3c shows a 2.5-Mb amplification event impacting the RET gene on chromosome 17. This SV was detected by PacBio, Illumina, and 10X Genomics linked read technologies and confirmed by Bionano mapping and Affymetrix array. There was a larger proportion of SVs validated by only one approach but not by other methods, demonstrating the strength of applying multiple orthogonal methods to confirm high-complexity SV events. In total, we confirmed 246 SVs from our consensus SV call sets and established our validation call set (Additional file 5: Table S5).

### SV annotation and oncogenic SVs in the reference samples

**Fig. 4:**
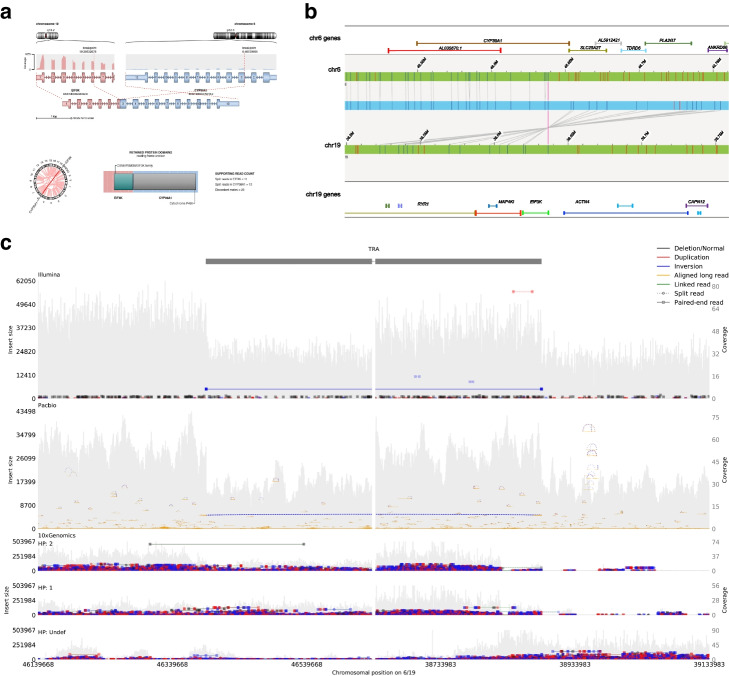
The inter-chromosomal translocation event for fusion gene EIF3K–CYP39A1 detected and validated by multiple technologies. **a** CYP39A1 gene on chromosome 6 is translocated and inverted to make a fusion transcript with EIF3K gene on chromosome 19. The fusion transcript was detected from RNA-seq data. **b** EIF3K–CYP39A1 translocation and inversion event detected from Bionano optical mapping. The blue bar in the middle denotes the reference genome, the green bars denote the optical mappings and the vertical lines between the blue and green bars represent the mapping between reference and maps. Top green bar shows maps on chromosome 6 and the bottom green bar shows maps on chromosome 19. **c** EIF3K–CYP39A1 translocation and inversion event are detected from NGS technologies from Illumina (**top**), PacBio (**middle**), and 10X Genomics linked reads (**bottom**). The visualization is generated using SVVIZ

To investigate the functional consequences of SVs, we annotated consensus SVs using AnnoSV to rank the structural variants. We identified 53 SVs including deletions and duplication events which were either confirmed to be pathogenic or described as possibly pathogenic (classes 4 and 5, respectively) according to AnnoSV [29, 30] (Additional file 11: Table S10**)**. In addition, we identified 13 inversion and 11 translocation events associated with fusion transcripts. In examining regions affected by SVs, we noticed 186 of the deletions were involved in the promoter and/or distal enhancer region. Previous studies also revealed many human structural variations are located in proximity to genes known to be mutated in important pathways in cancer biology [31]. We extracted 122 cancer-related genes which overlapped with the consensus SVs (Additional file 12: Table S11). Highly mutated genes like BARD1, MALT1, BRCA1/BRCA2, EIF3K, PTEN, FGFR2, and MAP3K1 were also found to overlap with SVs of the tumor sample that were identified by one or more technologies in our study (Fig. 4). The fusion gene events included ICMT-FPR153 fusion event, which is a known cancer-associated transcript fusion [32]. We also identified EIF3K–CYP39A1 among the cancer-related fusion genes, which is a known fusion transcript in breast cancer, that has been reported by previous studies [33, 34]. This translocation event was detected by three platforms: Illumina, PacBio, and 10X Genomics linked reads and confirmed by Bionano optical mapping (Fig. 4). Many of the large SVs are complex events consisting of several smaller SVs involved in the same region. We observed 23 genes among the 122 cancer-related genes involved in two or more structural variants. Additional file 10: Fig. S4 shows one 16 Mb duplication event detected by Bionano map on chromosome 17 of the cancer genome with breakpoints coinciding with translocations t(3;17) and t(12;17). This duplication event impacts multiple genes such as USP6, RABEP1, TP53, PER1, GAS7, MAP2K4, NCOR1, and FLCN, which implies that the duplicated copy of the genes might be fused with chromosomes 3 and 12. A previous study [35] described such events as “multi-hop” gene fusions, and these can be commonly observed in cancer genomes. We also observed a complex event known as chromosomal shattering, or chromothripsis, in our Hi-C data set. This is an event thought to occur early in cell development that has the potential to influence cancer cell progression [36, 37] (Additional file 10: Fig. S5). Furthermore, we ran Reconstructing Cancer Karyotypes (RCK) [20] software to infer the clone- and haplotype-specific cancer genome karyotypes based on the SVs and CNVs from the consensus call set, the result (Fig. 3e) revealed the cancer genome heterogeneity and is consistent with our previous study for this cell line [21, 22].

### Tumor purity impact for SV detection sensitivity

**Fig. 5:**
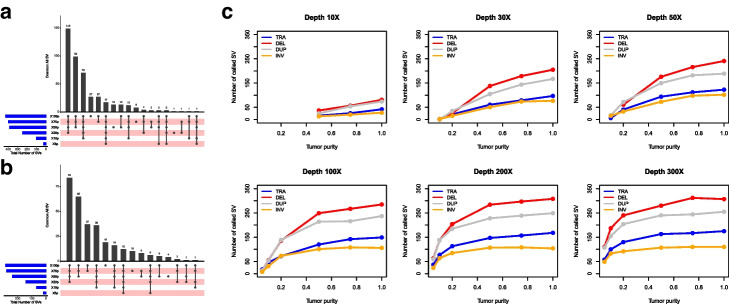
Effect of tumor purity and sequencing depth for SV detection. **a** Comparing small SV (50bp–30kb size range) detection sensitivities in different tumor purities in 5, 10, 20, 50, 75, and 100% tumor purity with 100x sequencing coverage. **b** Comparing large SV (>30kb) detection sensitivities in different tumor purities in 5, 10, 20, 50, 75, and 100% tumor purity at 100x sequencing coverage. The blue horizontal bars on the left show the total number of SVs in the specific tumor purity, pink bar with black dots denote number of SVs called in each tumor purity data. The top black vertical bars display the total concordance calls among the different tumor purities. **c** Line charts display SV detection sensitivity among different sequencing depth (10X, 30X, 50X, 100X, 200X, 300X) and different tumor purities (5%, 10%, 20%, 50%, 100%). The reference call set was built with consensus methodology and used the SVs from our consensus call set. The recalled SVs were separated into TRA (Translocation), DEL (Deletion), DUP (Duplication), and INV (Inversion)

To evaluate the effect of the amount of normal cell admixture (tumor purity) and sample heterogeneity, we mixed tumor and normal samples by spiking-in tumor DNAs into normal DNAs in the ratio of 5, 10, 20, 75, and 100% tumor (Additional file 13&14: Table S12 & S13). We pooled reads from Illumina WGS triplicate runs on samples that were sequenced at 100X coverage to generate coverage of 300X on each cell line, the data sets were subsampled to 300X, 200X, 100X, 80X, 50X, 30X, and 10X, and low sequencing depth datasets were included in high depth dataset. We compared the SV detection sensitivity based on tumor purity, sequencing depth, and SV types among the 16,858 SVs called by TNScope including 6431 large SVs and 10,453 small SVs (Fig. 5, Additional file 14: Table S13). We observed higher numbers of SV detection when tumor purity > 20% relative to lower tumor purity. When tumor purity was less than 20%, the detection sensitivity largely decreased. We also compared the combined effect of both tumor purity and sequencing depth on SV detection sensitivity. When tumor purity was greater than 50%, the percentage of SVs detected was above 50% for a sequencing depth as low as 30X. However, when tumor purity was low, it required much higher sequencing depth. The detection sensitivities and sequencing depth was very well correlated. Our results indicated the performance of SV detection was robust under conditions of moderate sample heterogeneity.

### Assessment of relative sensitivity for SV calls across platforms

To evaluate the performance of different platforms, we compared the SVs called by PacBio, Nanopore, Illumina, Bionano, 10X Chromium, and Hi-C including SVs from our initial combined call set defined in Additional file 3: Table S3 and Bionano call set from Additional file 15: Table S14. We measured the inter-platform relative sensitivity (Additional file 10: Fig. S6) by computing the proportion of all calls made by a reference platform that was reproduced by another technology. Overall relative sensitivity (Additional file 10: Fig. S6b) indicated a high concordance between Nanopore and PacBio SV calls (>75% PacBio calls are reproducible from Nanopore calls). Illumina SV calls showed moderate concordance with 10X and Bionano while 10X and Bionano showed limited power to recapitulate Illumina SV calls. Hi-C SV calls in our initial call set were redundant (>80% for Illumina and 10X) with all platforms, which was partially due to Hi-C Selva unique calls were filtered as the initial consensus call set required at least 2 software tools or 2 replicates making the same call. Stratifying relative sensitivity of one technology recover SVs from “reference” SV call set by SV size (Additional file 10: Fig. S6c & 6d), we found that Illumina short-read technology predicted fewer than 25% of PacBio and Nanopore SV calls smaller than 1kbp and predicted over 75% of PacBio and Nanopore SV calls for the SVs between 5 and 500kpb. Conversely, PacBio and Nanopore predicted close to 50% of Illumina SV calls regardless of the SV size. Illumina, PacBio, and Nanopore provided the most reproducible calls for small and moderate size SVs, whereas Bionano and 10x calls appeared to be more similar, i.e., up to 50% of Bionano calls could be predicted by 10X between 50 and 500kb while over 45% of 10X SV calls larger than 1Mb could be predicted by Bionano. 10X predictive power appeared to peak for SV calls larger than 100kb yet 10X could not predict most Bionano SV calls (relative sensitivity <30%) regardless of size. Interestingly, Bionano SV calls for the SVs larger than 500kb were the hardest to predict and therefore the most unique from any platform (relative sensitivity < 50%). However, the predictability of similarly sized 10X SV calls increased for larger SV calls as Bionano predictability decreased. While we note that Nanopore and PacBio discovered fewer than 25% of small Illumina SV calls, this is less related to the orthogonality of the platforms than the large number of Illumina SV calls and the relatively small number of those SV calls reproduced by PacBio or Nanopore.

## Discussion

By leveraging the combination of multiple technologies and deep sequencing of paired breast cancer and normal cell lines, we systematically investigated SV detection, and developed an integrated SV call set. By comparing multiple replicates, multiple technologies/platforms, and five different software tools, we were able to establish a high-confidence consensus call set for the reference cancer sample. This set was composed of 1788 SVs including deletion, duplication, insertion, inversion, and translocation events. Our study revealed that the choice of NGS technology was one of the major factors determining SV discovery accuracy and sensitivity. However, software algorithm contributed to the SV detection accuracy as well. While we observed low somatic SV call concordance across different technologies and software tools, there was consistency for specific SV types and sizes (Fig. 2, Additional file 10: Fig. S6). Smaller SV calls were more consistent across bioinformatics tools and NGS technologies than the larger SVs. Comparing to small deletion events detected by Illumina, PacBio, and the 10X Genomics platform, we noticed that Illumina detected a higher proportion of deletions in the 500bp–1kb range in initial call set. All three platforms detected much higher confidence of the small deletions in the 50bp–100bp range (Fig. 2a, Fig. 3c, Additional file 10: Fig. S6). For SVs that were kilobases to megabases in length, we observed PacBio, Nanopore, and Hi-C had higher concordance than short-read and linked read technologies. For Bionano SV calls, of the total 678 somatic SVs called in at least in 2 tumor sample replicates, 240 were corroborated by at least three other platforms, indicating the high fidelity of Bionano calls, and yet, only 43 of the 241 large Bionano SV calls were corroborated by at least two other platforms (Additional file 15: Table S14, Additional file 10: Fig. S6**)**. In fact, Bionano SV calls larger than 50kb were the least discoverable calls of any platform (Additional file 10: Fig. S6). Smaller SV calls were highly consistent with other platforms, suggesting that the Bionano platform is robust and consistent, and the uniqueness of large Bionano SV calls made them difficult to verify from another platform. In addition, we observed that the long-read technologies uncovered more complex SVs as well as large SVs, and those events tend to be missed or miscalled by short-read technologies.

Large translocation events between chr6:60,243,000 and chr16:34,260,000 were detected in the normal B lymphocyte transformed cell line (Additional file 10: Fig. S7), which involved genes including HLA-B, MIR6891, LOC101929072, MICA, LINC01149, HCP5, HCG26, MICB, MCCD1, and DDX39B on chr6 and VPS35, ORC6, MYLK3, and GPT2 on chr16. While both Illumina and PacBio missed those calls, both Hi-C and 10X Genomics reported only one of translocation events that was part of the chr6 p22 and chr16 q21 large inter-chromosomal translocation complex event. We also observed that the choice of software algorithm represented an important factor for SV detection accuracy. Some software tools were more consistent with each other. For example, PBSV, Sniffles, and NanoSV had high overlap when used for PacBio and Nanopore SVs calls (Fig. 2c, Additional file 10: Fig. S8). Similarly, Manta, novoBreak, and TNscope were highly concordant when used for Illumina short-read SV calls. On the other hand, Long Ranger and novoBreak had many tool-specific “unique” calls compared to the other callers. Delly seemed to have the fewest concordant calls with other comparable software tools. Promisingly, each tool seemed to have its own strength in certain SV types. For example, PBSV was robust for small deletions, Sniffles performed well on inversions, while Selva identified most of the complex events, likely due to the Hi-C technology (Fig. 2a, Fig. 3c). Strikingly, aligner choice seemed to have little impact, and the results from different aligners (BWA and NovoAlign) were highly concordant (Additional file 10: Fig. S1**).**

Due to the technical limitations of each technology and computational challenges associated with the complex SV detection, our study has inherent limitations. First of all, the HCC1395 cell line’s structural variant profile and complexity may not be representative of other cancer types. In addition, primary tumor samples are commonly heterogenous, and detecting low allele frequency of structural variants in subclonal populations of tumor cells can be challenging. The other limitation from our study is the lack of a truth set for comparing the software tool’s detection sensitivity and accuracy. To address this limitation to the greatest extent possible, our study has utilized a combination of short-read and long-read technologies, as well as DNA and RNA sequencing for SV discovery and confirmation with deep sequencing, which has helped us elucidate many structural variants in this cancer genome. Our high-confident call set requires at least two technologies to agree on the same structural variant based on SV types and SV sizes, while the stringent protocol works better for establishing a high-quality call set, but it is at the expense for detection sensitivity. Due to the current technology and software algorithm limitations, some of the complex break point events were not consistently called across technologies and need to be further characterized (Additional file 10: Fig. S9, Additional file 16: Table S15). We acknowledge that there is a need for continuous improvement of the SV call set for the HCC1395 cancer genome as new technologies and more accurate SV calling algorithms and pipelines become available.

## Conclusions

Exciting recent research has detailed the importance of SVs for clinical practice, cancer molecular biology, pharma R&D, and more. However, high-throughput rigorous and robust SV calling is not yet available as a standard practice. In this study, we have investigated the strengths and limitations of each technology and software tools in the context of SV detection. Our findings indicate that the community should use multiple tools and a consensus approach to reduce false positives when studying SVs. Our study showed that an integration of multiple technologies could improve SV calling sensitivity and accuracy for the cancer genome. The methods, actionable insights, and datasets presented in this study will serve as a valuable resource to the scientific community for future genome sequencing structural variant studies.

## Methods

### Cell line and DNA extraction

Genomics DNA was prepared by ATCC using cell expansions from master banks of cells for the HCC1395 (ATCC, CRL-2324) and HCC1395BL (ATCC, CRL-2325) cell lines. Both cell lines were validated at ATCC using multiple cell-specific markers for each cell line. The karyotyping is described in “Cell line karyotyping” section.

Homo Sapiens Breast Carcinoma HCC1395 cells (expanded from ATCC CRL-2324) were cultured in ATCC-formulated RPMI-1640 Medium (ATCC 30-2001) supplemented with fetal bovine serum (ATCC 30-2020) to a final concentration of 10%. Cells were maintained at 37 °C with 5% carbon dioxide (CO_2_) and were subcultured every 2 to 3 days, per ATCC recommended procedures using 0.25% (w/v) Trypsin-0.53 mM EDTA solution (ATCC 30-2101), until appropriate densities were reached. Additionally, an Epstein-Barr virus (EBV) transformed B-lymphoblast cell line (HCC1395BL) (expanded from ATCC CRL-2325) was cultured in ATCC-formulated Iscove’s modified Dulbecco’s medium (ATCC Catalog No. 30-2005) supplemented with fetal bovine serum (ATCC 30-2020) to a final concentration of 20%. Cells were maintained at 37 °C with 5% CO_2_ and were subcultured every 2 to 3 days, per ATCC recommended procedures, using centrifugation with subsequent resuspension in fresh medium until appropriate densities were reached. Final cell suspensions were spun down and resuspended in PBS for nucleic acid extraction.

All cellular genomic material was extracted using a modified Phenol-Chloroform-Iso-Amyl alcohol extraction approach. Essentially, cell pellets were resuspended in TE, were subjected to lysis in a 2% TritonX-100/0.1% SDS/0.1 M NaCl/10mM Tris/1mM EDTA solution, and were extracted with a mixture of glass beads and Phenol-Chloroform-Iso-Amyl alcohol. Following multiple rounds of extraction, the aqueous layer was further treated with Chloroform-IAA and finally underwent RNAse treatment and DNA precipitation using sodium acetate (3 M, pH 5.2) and ice-cold ethanol. The final DNA preparation was resuspended in TE and stored at −80°C until use.

### Cell line karyotyping

Karyotyping was performed by Cell Line Genetics (Madison, Wisconsin) essentially as described previously [38]. Cells were treated with Colcemid (Gibco) for 40 min followed by exposure to 0.075M KCl for 23 min at 37°C and then fixed with 3:1 methanol:glacial acetic acid. Slides were stained with Leishman’s stain before observation. During observation, roughly 20 metaphase cells were counted by microscope and numerical and structural chromosome aberrations were recorded. An analysis of 5–10 cell bands was performed at the microscope with a ×100 objective, with an effort to karyotype at least two cells from each clone.

### Illumina WGS library preparation

The TruSeq DNA PCR-Free LT Kit (Illumina, FC-121-3001) was used to prepare samples for whole-genome sequencing. WGS libraries were prepared at six sites with the TruSeq DNA PCR-Free LT Kit according to the manufacturers’ protocol. One microgram of DNA was used for the TruSeq-PCR-free libraries, unless otherwise specified. All sites used identical fragmentation conditions for WGS by using Covaris with a 350-bp target size. All WGS replicates were prepared on a different day. The input amount of WGS runs with fresh DNA was 1 μg unless otherwise specified.

The concentration of the TruSeq DNA PCR-Free libraries for WGS was measured by qPCR with the KAPA Library Quantification Complete Kit (Universal) (Roche, KK4824). The concentration of all the other libraries was measured by fluorometry either on the Qubit 1.0 fluorometer or on the GloMax Luminometer with the Quant-iT dsDNA HS Assay kit (Thermo Fisher Scientific, Q32854). The quality of all libraries was assessed by capillary electrophoresis either on the 2100 Bioanalyzer or TapeStation instrument (Agilent) in combination with the High Sensitivity DNA Kit (Agilent, 5067-4626) or the DNA 1000 Kit (Agilent, 5067-1504) or on the 4200 TapeStation instrument (Agilent) with the D1000 assay (Agilent, 5067-5582 and 5067-5583).

For the tumor purity study, 1 μg tumor:normal dilutions were made in the following ratios using Resuspension Buffer (Illumina): 1:0, 3:1, 1:1, 1:4, 1:9, 1:19, and 0:1. Each ratio was diluted in triplicate. DNA was sheared using the Covaris S220 to target a 350-bp fragment size (peak power 140W, duty factor 10%, 200 cycles/bursts, 55s, temp 4 °C). NGS library preparation was performed using the Truseq DNA PCR-free protocol (Illumina) following the manufacturer’s recommendations.

Whole-genome libraries were sequenced on a HiSeq 4000 instrument (Illumina) at 2 × 150bp read length with HiSeq 3000/4000 SBS chemistry (Illumina, FC-410-1003), and on a NovaSeq instrument (Illumina) at 2 × 150bp read length using the S2 configuration (Illumina, PN 20012860). Sequencing was performed following the manufacturer’s instructions.

### 10X Genomics library preparation

10X Genomics library prep was performed following the 10X Genomics protocol which involves the generation of long-range information across the length of individual DNA molecules. Approximately 1.25–1.5 ng (~375–450 haploid genome equivalents) of template gDNA is required as input for the GEM Generation & Barcoding step. It is critical to quantify template HMW gDNA accurately to load the correct amount into the Sample Master Mix using the Qubit® Fluorometer system. The required concentrations of the diluted gDNA solution are 0.8–1.2 ng/μl before proceeding to preparing GEM Reagent Master Mix. Isothermal incubation of the GEMs produced barcoded fragments ranging from a few to several hundred base pairs. After incubation, the GEMs are broken and the pooled fractions are recovered. The read 1 sequence and the 10x™ Barcode are added to the molecules during the GEM incubation. The P5 and P7 primers, Read 2, and Sample Index are added during library construction via end repair, A-tailing, adaptor ligation, and amplification. The final libraries contain the P5 and P7 primers used in Illumina® bridge amplification.

### PacBio library preparation

Fifteen micrograms of material was sheared to 40kb with Megarupter (Diagenode). Per the Megarupter protocol, the samples were diluted to below 50ng/μl. A 1× AMPure XP bead cleanup was performed. Samples were prepared as outlined on the PacBio protocol titled “Preparing >30kb SMRTbell Libraries Using Megaruptor Shearing and BluePippin Size-Selection for PacBio RS II and Sequel Systems”. After library preparation, the library was run overnight to size select using the Blue Pippin (Sage). The Blue Pippin was set to select the size range of 15–50kb. After collection, a 1× AMPure XP bead cleanup was performed.

### Dovetail SELVA library preparation

Three Dovetail Hi-C libraries were prepared for each sample in a similar manner as described previously [39]. Briefly, for each library, chromatin was fixed in place with formaldehyde in the nucleus and then extracted. Fixed chromatin was digested with DpnII, the 5’ overhangs were filled in with biotinylated nucleotides, and then the free blunt ends were ligated. After ligation, crosslinks were reversed, and the DNA was purified from protein. Purified DNA was treated to remove biotin that was not internal to ligated fragments. The DNA was then sheared to ~350 bp mean fragment size and sequencing libraries were generated using NEBNext Ultra enzymes and Illumina-compatible adapters. Biotin-containing fragments were isolated using streptavidin beads before PCR enrichment of each library.

### NuGEN ovation universal RNA-seq preparation and sequencing

We isolated mRNA in bulk from HCC1395 and HCC1395 BL cells using the miRNeasy Mini kit (QIAGEN, 217004). The NuGEN Ovation universal RNA-seq kit was used for library preparation. Briefly, 100 ng of total RNA was reverse transcribed and then made into double-stranded cDNA (ds-cDNA) by the addition of a DNA polymerase. The ds-cDNA was fragmented to ~200 bp using the Covaris S220, and then underwent end repair to blunt the ends followed by barcoded adapter ligation. The remainder of the library preparation followed the manufacturer’s protocol. All the libraries were quantified with a TapeStation 2200 (Agilent Technologies) and a Qubit 3.0 (Life Technologies). We sequenced the libraries on a NextSeq550 with 75 bp paired end sequencing and on a HiSeq4000 with 100 bp paired end sequencing.

### DNA extraction and MinION sequencing

For HCC1395BL cells, Miltenyi dead cell removal kit (MACS Miltenyi Biotech, #130-090-101) was used to remove dead cells by following the manufacturer’s protocol. Briefly, HCC1395BL cells were pelleted and the supernatant was aspirated completely. Dead cell removal beads were used to resuspend the cells (100 μl per 1 × 10^7^ total cells). After incubation at room temperature for 15 min, the mixture was loaded onto Miltenyi LS column (MACS Miltenyi Biotech, #130-042-401) in 3 ml 1X binding buffer. Ten milliliters 1X binding buffer was used to elute/rinse the column following the passage of the cell/bead mixture. The flow through containing live cells was collected and assessed using an automated cell counter (Countess II, Thermo Fisher). The cell viabilities were measuring above 95% after the dead cell removal procedure. HCC1395 cells were detached by Accutase (Innovative Cell Technologies, CA) followed by three washes with PBS. The cell viability was greater than 90% without using Miltenyi dead cell removal kit.

Genomic DNA from HCC1395 and HCC1395BL cell lines was extracted using QIAGEN MagAttract HMW DNA Kit (QIAGEN, Hilden, Germany). One microgram of initial DNA without fragmentation was used for library construction using SQK-LSK109 ligation sequencing kit (Oxford Nanopore Technologies, Oxford, UK). All library preparations were conducted as per the protocols provided by ONT with the exception of the end-prep step where samples were incubated for 20 min at 20 °C and 5 min at 65 °C. Each library was sequenced separately on an individual MinION FLO-MIN106D R9.4 flowcell. Prior to sequencing, flowcell pore counts were measured using the MinKNOW Platform QC script (Oxford Nanopore Technologies, Oxford, UK). About 300 ng of completed libraries was loaded as per instructions from ONT. Raw sequence reads were basecalled in real time via MinKNOW. Basecalled data passing quality parameters (qmean > 7) were converted to fastq. Only reads designated as pass were included in further analyses.

### Affymetrix CytoScan array validation

We obtained DNA from two reference cell lines from ATCC: (HCC1395, SCCRL2324_ D; HCC1395 BL, ATCC®SCCRL2325_D). DNA concentration was measured with Nanodrop (Life technology), and integrity was evaluated with TapeStation 4200 (Agilent). Two hundred fifty nanograms of gDNA was used to proceed with the Affymetrix CytoScan Assay kit (Affymetrix). The workflow consisted of restriction enzyme digestion with Nsp I, ligation, PCR, purification, fragmentation, and end labeling. Then DNA was hybridized for 16 h at 50°C on CytoScan array (Affymetrix), followed by washing and staining in the Affymetrix Fluidics Station 450 (Affymetrix). Scanning was performed with the Affymetrix GeneChip Scanner 3000 G7 (Affymetrix). CytoScan Array CEL files were processed and analyzed with Affymetrix Chromosome Analysis Suite (ChAS, Affymetrix, Inc.). Array-specific annotations (NetAffx annotation release 36, built with human hg38 annotation, NetAffxGenomicAnnotation s.Homo_sapiens.hg38.na2 0170803.db) were used in analysis workflow module of ChAS. Karyoview plot and segment data were then exported in the ChAS browser with default parameters: losses ≥ 25 markers, gains ≥ 50 markers.

### PCR-based SV validation

For further validation of CNVs and other types of structural variants (balanced translocations, tandem duplications, inversions), PCR was used to target the breakpoints associated with a subset of putative SVs. qPCR was also used as an additional means of quantifying duplication events that could not be definitively described as tandem. Target loci were chosen to broadly represent the size ranges observed for each of the SV types, as well as the consensus scores associated with them in the case of SVs detected in multiple datasets. PCR products from the cancer cell line were compared to the normal cell line to identify cancer-specific variants.

All primers were designed using Primer3 v4.1 (http://primer3.ut.ee/) to limit oligo lengths to 20–27bp, temperatures to 55–62°C, and product sizes to 250-450bp. All other settings were left as the defaults, and self and pair complementarity scores were minimized to zero where possible. Products were amplified on either a C1000 or S1000 thermal cycler (Bio-Rad) from 1 ng of sample DNA using Enhanced PCR Mix (EPM, Illumina) according to the manufacturer’s instructions. Amplification conditions were as follows: 95°C for 3min; 35 cycles of 30s @98°C, 30s @55–62°C, 1min @72°C; 5min @72°C; hold @8°C. Products were run on 2% E-gels (Invitrogen) for 17 min and visualized on an AlphaImager (ProteinSimple).

All primers’ designed targeted regions were free of repetitive elements and segmental duplications wherever possible. To capture deletions and translocations, primer pairs were selected in the region ±500bp of the breakpoint as identified in the cancer cell line. For validation of small (<3.5kb) deletions, products of different expected sizes were observed in the cancer vs normal cell line. Larger deletions and translocations showed the expected product only in the cancer cell line. To capture inversions, primer pairs were selected in the region ±500bp of either inversion breakpoint, with validated products being detected only in the cancer cell line. To capture duplications, multiple regions were selected within the duplicated locus and amplified by endpoint PCR as described above to ensure the expected product was observed in both the cancer and normal cell lines. The primer pairs that clearly generated the expected products were then used for qPCR amplification of the target to determine the difference in quantitation cycles (dCq) between the cancer cell line and the normal cell line. In some instances, copy number differences could also be visualized qualitatively by endpoint PCR.

When the approach described above failed to generate the expected PCR products, alternate primers were designed to target the unaltered locus in the normal cell line, rather than the site of the breakpoint in the cancer cell. In these instances, diminished product was expected in the cancer cell line compared to the normal cell line and was verified by qPCR when not readily observed by endpoint PCR.

### Optical genome mapping for SV validation

Ultra-high molecular weight (UHMW) DNA was extracted from cryopreserved cells in frozen medium containing DMSO following the manufacturer’s protocols (Bionano Genomics, USA). Cells were digested with Proteinase K and RNAse A in a lysis binding buffer containing detergents. DNA was precipitated with isopropanol and bound with nanobind magnetic disk. Bound UHMW DNA was resuspended in the elution buffer and quantified with Qubit dsDNA assay kits (Thermo Fisher Scientific).

DNA labeling was performed following manufacturer’s protocols (Bionano Genomics, USA). Standard Direct Labeling Enzyme 1 (DLE-1) reactions were carried out using 750 ng of purified ultra-high molecular weight DNA. The fluorescently labeled DNA molecules were counter-stained and imaged across nanochannels on a 2nd-generation Saphyr instrument. Genomic coverage of approximately 400X was achieved for all tested samples.

Genome analysis was performed using software provided by Bionano Genomics (Bionano Solve [40] 3.5, Access 1.5). Rare Variant Analysis was performed to sensitively capture somatic SVs occurring at low allelic fractions. Briefly, molecules of a given sample dataset were first aligned against the public Genome Reference Consortium GRCh38 human assembly. SVs were identified based on discrepant alignment between sample molecules and GRCh38, with no assumption about ploidy. Consensus genome maps (*.cmaps) were then assembled from clustered sets of molecules that identified the same variant. Finally, the CMAPs were realigned to GRCh38, with SV data confirmed by consensus forming final SV calls.

Finally, fractional copy number analysis was performed from alignment of molecules and labels against GRCh38 (alignmolvref). A sample’s raw label coverage was normalized against relative coverage from normal human controls, segmented, and baseline CN state estimated from calculating mode of coverage of all labels. If Y Chromosome molecules were present, baseline coverage in sex chromosomes was halved. With a baseline estimated, CN states of segmented genomic intervals were assessed for significant increase/decrease from the baseline. Corresponding duplication and deletion copy number variant calls were output. Certain SV and CN calls were masked, if occurring in GRC38 regions found to be high variance (gaps, segmental duplications, etc.)

### DNA sequencing

#### Illumina sequencing

Illumina whole-genome libraries were sequenced on a HiSeq 4000 instrument (Illumina) at 2 × 150 bp read length with HiSeq 3000/4000 SBS chemistry (Illumina, FC-410-1003), and on a NovaSeq instrument (Illumina) at 2 × 150 bp read length using the S2 configuration (Illumina, PN 20012860).

The sequencing depth was ~800M read pairs per library for a total 21 libraries.

FASTQ sequence files for whole-genome and whole-exome sequencing were generated from the Illumina sequencer images using the Illumina RTA 1.18.66.3 (HiSeq 2500) or 2.7.7 (HiSeq 4000) and bcl2fastq 2.17.1.14 software.

#### PacBio sequencing

The samples were loaded on the PacBio Sequel (Pacific Biosciences) following the protocol titled “Protocol for loading the Sequel.” The recipe for loading the instrument was generated by the Pacbio SMRTlink software v 5.0.0. Libraries were prepared using Sequel chemistry kits v 2.1, SMRTbell template kit 1.0 SPv3, magbead v2 kit for magbead loading, sequencing primer v3, and SMRTbell cleanup columns v2. Libraries were loaded at between 4 and 8pM.

#### 10X Genomics sequencing

The 10X Genomics libraries contained the P5 and P7 primers which are compatible with Illumina sequencing platforms and are used in Illumina bridge amplification. The final purified product was then quantitated by qPCR before cluster generation and sequencing. The sequencing was done on the HiSeq4000 instrument using a 2×150bp pair-end run. The sequencing depth was ~940M read pairs per library for a total 22 libraries.

#### Hi-C sequencing

The Dovetail Hi-C libraries were loaded and sequenced on an Illumina HiSeq X with a pair-end run setup as 2×75bp. The sequencing depth is ~220M read pairs per library for a total of six libraries.

### Bioinformatics pipelines

In this section, we described the step-by-step bioinformatics analysis pipelines that were used to call the SVs from each method and integrate the multiple call sets to obtain the somatic SV high-confidence call set. The bioinformatics workflow is shown in Fig. 1b. The exact commands are documented in the specific section for each task.

### Reference genome

The reference genome we used was the decoy version of the GRCh38/hg38 human reference genome (https://gdc.cancer.gov/about-data/data-harmonization-and-generation/gdc-reference-files; GRCh38.d1.dv1.fa), which was utilized by the Genomic Data Commons (GDC). The gene annotation (GTF) file was downloaded from the 10X Genomics website as refdata-cellranger-GRCh38-1.2.0.tar.gz, which corresponds to the GRCh38 genome and Ensembl v84 transcriptome. All of the following bioinformatics data analyses are based on the above reference genome and gene annotation.

### Illumina WGS read preprocessing and alignment

Illumina bcl2fastq2 (v2.17) [41] was used to demultiplex and convert binary base calls and quality scores to FASTQ format. FASTQC (v0.11.2 ) [42] was run on the raw reads to assess basecall quality, adapter content, G/C content, sequencing length, and duplication level. In addition, FASTQ_screen (v0.5.1) and miniKraken (v0.10.0) [43] were run to detect possible cross contamination with other species. A multiQC (v1.3) [44] run report was generated for each sample set. The sequencing reads were trimmed of adapters and low-quality bases using Trimmomatic (v0.30) [45]. The trimmed reads were mapped to the human reference genome GRCm38 (see the read alignment section) using BWA-mem (v0.7.12) [26] in paired end mode. In addition, the DNA Damage Estimator (v3) [46] was used to calculate the GIV score based on an imbalance between R1 and R2 variant frequency of the sequencing reads to estimate the level of DNA damage that was introduced in the sample/library preparation processes. Post alignment QC was performed based on BWA alignment BAM files, the genome mapped percentages and mapped read duplication rates calculated by BamTools (v2.2.3) [47] and Picard (v1.84) [48]. The genome coverage and exome target region coverages as well as mapped reads insert sizes, and G/C contents were profiled using Qualimap (v2.2) [49] and custom scripts. Preprocessing QC reports were generated during each step of the process. MultiQC (v1.3) [44] was run to generate an aggregated report in html format. A standard QC metrics report was generated from a custom script. For all alignments, we used BWA-MEM v0.7.17 [26] with the –M flag for downstream Picard compatibility.

### Illumina short-read SV calling

SVs were detected by four independent pipelines. Illumina short reads (untrimmed) from 11 pairs of tumor/normal samples were aligned onto GRCh38 reference genome using bwa-mem (version 0.7.17-r1194) with default parameters to a GRCh38 human reference genome (see reference genome section above). All the alignment bam files were duplicate-marked by Picard before SV calling. Reads with a mapping quality of at least 20 were retained for SV detection.

In the first pipeline, SV calls were generated from this mapped data using Delly [50] (Version: 0.7.8) with default parameters was used to generate 11 Delly SV call sets following the procedures described in Delly’s Github repo (https://github.com/dellytools/delly) for somatic SV calling. Delly detects deletions, inversions, tandem duplications, insertions, and inter-chromosomal translocations.

In the second pipeline, SV calls were generated from this mapped data using NovoBreak [51] (version v1.1.3rc). Default parameters were used to generate 11 NovoBreak SV call sets following the detailed procedures were depicted at the tool’s GitHub repo (https://github.com/czc/nb_distribution).

In the third pipeline, TNscope [52] (v201711.03) was used to process the BWA-MEM alignment BAM file to call somatic SVs, and DNAscope (v201711.03) was used to call germline SVs.

For TNscope (v201711.03), we used the version implemented in Seven Bridges’s CGC. Sentieon TNscope is a somatic variant caller for SNV, Indel, and SV. Here, we use TNscope only for SV calling. TNscope identifies statistical confidence breakends that only occur in the tumor sample.

*Command:*

*sentieon driver -r GRCh38.d1.vd1.fa -i $tumor.bam -i $normal.bam --algo TNscope --tumor_sample $tumor_sample_name --normal_sample $normal_sample_name --disable_detector snv_indel $output.vcf.gz.*

Sentieon DNAscope is a germline variant caller for SNV, Indel, and SV. Here, we use DNAscope only for SV calling. DNAscope identifies statistical confident germline breakends. We used the version 201711.03 implemented in Seven Bridges’s CGC with the following commands:

*Command:*

*sentieon driver -r GRCh38.d1.vd1.fa -i $tumor.bam -i $normal.bam --algo DNAscope --var_type bnd tmp.vcf.gz && sentieon driver -r GRCh38.d1.vd1.fa --algo SVSolver -v tmp.vcf.gz $output.sv.vcf.gz*

The fourth pipeline was performed using the Manta [53] integrated in the Dragen pipeline [27]. The BWA-MEM alignment BAM files were used to call somatic SVs as follows:

*Command:*

*dragen -f -r GRCh38.d1.vd1_hash --sv-reference GrCh38.d1.vd1.fa --output-file-prefix $tumor_vs_normal --tumor-bam-input $tumor.bam --bam-input $normal.bam --enable-map-align false --enable-map-align-output false --enable-sv true*

### 10X Genomics linked read SV analysis

The 10X Genomics Chromium fastq files were mapped and reads were phased using LongRanger [54] to the hg38/GRCh38 reference genome using the LongRanger v2.2.2 pipeline [https://genome.cshlp.org/content/29/4/635.full]. The linked reads were aligned using the Lariat aligner [55] [https://genome.cshlp.org/content/29/4/635.full], which uses BWA [26] to generate alignment candidates, and duplicate reads are marked after alignment. SV calls that were within 10 kb of gaps or new sequences introduced in GRCh38 are also filtered because such calls likely represent misassemblies in hg19.

The Manta methods integrated in the Dragen pipeline were also used to call SVs from the 10X Genomics Chromium fastq files. The alignment BAM files were generated by the Dragen pipeline, and then used for somatic SV calling as follows:

*Command:*

*dragen -f -r GRCh38.d1.vd1_hash --tumor-fastq-list-sample-id $tumor --tumor-fastq-list fastq_list.csv --fastq-list-sample-id $normal --fastq-list fastq_list.csv --enable-duplicate-marking true --enable-variant-caller true --output-file-prefix $tumor_vs_$normal --enable-map-align-output true --enable-bam-indexing true --dbsnp dbsnp_146.hg38.vcf.gz --cosmic COSMIC_82_hg38.vcf.gz --panel-of-normals 1KG.2504.plus.TCGA.ACgrt0.vcf.gz --bin_memory=60000000000*

*dragen -f -r GRCh38.d1.vd1_hash --sv-reference GRCh38.d1.vd1.fa --output-file-prefix $tumor_vs_normal --tumor-bam-input $tumor.bam --bam-input $normal.bam --enable-map-align false --enable-map-align-output false --enable-sv true*

### PacBio single-molecule long-read SV analysis

#### NGM-LR and Sniffles

The raw bam files were merged per sample and then were aligned using smrttools v5.0.1 software which includes PBSV. PBSV utilized NGM-LR [56] as default aligner and used hg38/GRCh38 as reference genome. The PBSV align commands also marked or removed the duplicated read bases on the reads coming from the same ZMW, the base pair tolerance was set to 100bp to remove the duplicated reads.

The resulting bam file was used in Sniffles [56] to call SV per sample from the PacBio data using the following command:

*Command: sniffles -m tumor.merge.ngmlr.dedup.bam -v tumor.merge.ngmlr.dedup.vcf -t 16 --tmp_file ./ -l 30*

#### Minimap2 and PBSV

Raw merged bam files were aligned using smrttools v6.0.1. pbmm2 which used minimap2 [57] as default aligner. SVs were called using PBSV discover and call command per sample.

*pbmm2 align --sort --sort-memory 24G --total-threads 24 --sort-threads-perc 30 --preset SUBREAD normal.merge.raw.bam hg38.fa normal.pbmm2.bam*

*pbsv discover --log-file discover.log -s normal normal.pbmm2.bam normal.svsig.gz*

*pbsv call --log-file call.log -j 24 hg38.fa normal.filter.svsig.gz normal.pbsv.smrtv6.vcf*

#### Somatic SVs from PacBio

The resulting variant vcf file was divided into two files: large SVs (translocations and SV >30kb) and small SVs (no translocation and SV <30kb). Analysis using Survivor was performed similarly as for 10X Genomics large SVs and small deletions to get somatic large and small SVs.

### Oxford Nanopore sequencing data SV analysis

Basecalls were performed using Guppy v3.3.3. Data passing quality parameters (qmean > 7) were converted to fastq. The raw fastq files were merged for each sample and then were mapped to hg38 reference using mimimap v2-2.16.

*minimap2 -ax map-ont --MD hg38.fa.fastq.gz | samtools view -bS - | samtools sort -m 16G -@16 - > tumor.ont.bam*

Mapped bam files were used to call SVs using Sniffles v1.0.11 and Nanosv v1.2.4. Sniffles was run with -s 3 -l 50 -t 36 --report_BND parameters:

*sniffles -s 3 -l 50 -t 36 --report_BND -m tumor.ont.bam -v tumor.ont.sniffles.vcf*

Nanosv was run using default config parameters:

*NanoSV -t 36 -o tumor.ont.nanosv.vcf -c config.txt -b default.bed tumor.ont.bam*

### Dovetail SELVA data SV analysis

A pair of normal and tumor libraries were sequenced to a depth of 34X and 37X respectively on a Illumina NextSeq for gene fusion identification. The ideal sequencing depth was determined via estimation of library complexity from a MiSeq QC process.

Reads were aligned to the GRCh38 human reference genome. The mapping pipeline maps each read separately using the aligner BWA-MEM [26]. BWA-MEM maps paired end data separately as two single end reads due to the potentially long separation distance between paired end reads. We combined two single end reads as a single paired end read in the BAM file and removed unmapped reads through a post processing step and used Picard tool to remove PCR duplicates.

Chromosomal rearrangements and gene fusions were assessed by dividing the reference genome into non-overlapping bins of width *w*, and tabulating *N*_*ij*_ the number of read pairs which map with high confidence (MAPQ > 20) to bins *i* and *j* respectively.

To automatically identify genomic rearrangement junctions, we defined a statistic that identifies local contrasts in *N*_*ij*_ characteristic of rearrangements. Assuming Poisson-distributed local read counts, we computed two *z*-scores at each bin *i,j*: ${Z}_{ij}^{+}=\frac{\left({N}_{ij}^{+}-{N}_{ij}^{-}\right)}{\sqrt{N_{ij}^{-}}}$ and ${Z}_{ij}^{-}=\frac{\left({N}_{ij}^{-}-{N}_{ij}^{+}\right)}{\sqrt{N_{ij}^{+}}}$

Where $${N}_{ij}^{+}$$ is the local sum over north-east and south-west quadrants of *N*_*ij*_ up to a maximum range *R*: $${N}_{ij}^{+}=\sum_{k=i,l=j}^{k=i+R,j+R}{N}_{kl}+$$$$\sum_{k=i,l=j}^{k=i-R,j-R}{N}_{kl}$$, and $${N}_{ij}^{-}$$ is a similar sum over north-west and south-east quadrants: $${N}_{ij}^{-}=\sum_{k=i,l=j}^{k=i-R,j+R}{N}_{kl}+$$$$\sum_{k=i,l=j}^{k=i+R,j-R}{N}_{kl}$$. All positions *ij* for which max($${Z}_{ij}^{+}$$_,_$${Z}_{ij}^{-}$$) > *Z*_min_= 1 and max($${Z}_{ij}^{+}$$_,_$${Z}_{ij}^{-}$$) is a local maximum (no positions *i*,*j* have a higher value within a range of 3w) were defined as candidate fusion junctions.

After identifying candidate fusions at an initial bin size *w*_0_ = 50,000, we classified candidate fusions using a convolutional neural network (CNN) model [58] to identify positive and negative class. The probability of calling it a positive fusion was set to be above 0.7. After classifying, we refined the breakpoint position using two kernels with values decaying from the center as an exponential function. One kernel had values only on the north-east and south-west quadrants, and another kernel had values only on the north-west and south-east quadrants. Each kernel was convolved over a contact matrix that was centered at the candidate fusion and 2Mb to the left and right. After convolving, we calculated the global maxima of these two convolved images and selected the maximum as the refined breakpoint. Fusion event classification was done by selecting a new contact matrix with the same dimensions as above centered at the refined breakpoint and calculating 4 quadrant coverage. Depending on the quadrant values, fusion events are classified as a reciprocal translocation, non-reciprocal translocation, deletion, inversion, and segmental duplication.

### Bionano SV analysis

SV calls were performed using Bionano Saphyr (Access 1.5) [40]. A non-redundant set of Bionano calls (SVs and CNVs) are selected only if they appear in at least 2 of the 3 replicates. Those variants must be in tumor and not in the paired normal libraries which were selected against the consensus callset from other technologies by clustering analysis. The SVs called by other technologies and validated by Bionano both have the same call, and the two calls would be clustered together. The criteria to determine if the two sets can be clustered together:

1. Insertion/deletion: only look at calls > 500 bp in size due to Bionano detection resolution is 500bp:
  The two calls have similar size (>50% similarity) and two calls can be no more than 10kb apart
2. Inversion: only look at calls > 500 bp in size and require the two calls have similar size (>50% similarity) and the two calls can be no more than 10kb apart
3. Duplication: only look at calls > 500 bp in size and require the two calls have similar size (>50% similarity) and the two calls can be no more than 10kb apart. The direction of duplication (direct or inverted) is not considered, thus can be clustered together regardless of the direction
4. Translocation: the two calls’ breakpoints are not more than 50 kb apart and direction of translocation is not considered, thus can be clustered together regardless of the direction

### Multi-platforms SV integration

The somatic SV events such as insertions, deletions, intra-chromosomal inversions, inter-chromosomal translocations, and complex breakpoint events were called in a tool-specific manner in the above sections described in platform/tool-specific SV analysis. If somatic calling mode was not supported by a specific software, Survivor [25] v1.0.3 SVs was used to get SVs in tumor sample and not in the normal sample for each pair of the sample.

### Split VCF files

The SVs in each tool-specific VCF file were split based on the 5 window sizes (50 to100bp, 101 to 500bp, 501 to 1000bp, 1001 to 30,000bp, 30,000bp and above). Translocation events (TRA) were extracted from the split VCF file to save into a separate file for each window bin group. The Survivor filter function was used to split VCF files as follows*:*

*SURVIVOR filter $input.vcf NA $min_size $max_size 0 -1 $window_bin/$output.vcf && cat $window_bin/$output.vcf | grep -w -v "SVTYPE=TRA" | vcf-sort >$window_bin/$output.noTRA.vcf && cat $window_bin/$output.vcf | grep -w "SVTYPE=TRA" | vcf-sort >$window_bin/$output.only.TRA.vcf*

#### Somatic large SV

To get the tumor-only (somatic) SVs, the large SV (SV >30kb) VCF files for each pair were merged using Survivor. The SVs were merged if they were <10kb between the break points. SVs which were only present in the tumor samples and not in the normal sample were taken as somatic SVs for each pair of the sample. The command used for merging the VCF files for SV integration by window sizes (10,000bp) was:

*Command: SURVIVOR merge $input.vcf.list 10000 1 0 0 0 10000 $output.vcf*

#### Somatic small SV

To get tumor-specific small deletions, insertions, duplications, and inversions, the VCF file per sample was split into 4 files based on the length of the SV size: 50 to 100bp, 101 to 500bp, 501bp to 1kB and SV (size) greater than 1kb but smaller than 30kb. Each of the pairs of samples was then merged using Survivor. Their minimum SV length was used as the maximum distance between the break point. The resulting tumor-specific SVs were then merged from all the four files as tumor-specific SVs for each pair. The command used for merging the VCF files for SV integration by window sizes (50, 100, 500, 1000) was:

*SURVIVOR merge $input.vcf.list $window_size 1 0 0 0 $window_size $output.vcf*

#### Merging somatic call sets

Large SVs (SV > 30kb or translocations) from the tumor-only filter calls from all samples from all the tools (11 LongRanger, 11 GrocSV [59], 11 TNScope,11 Novobreak, 11 Delly, 1 Sniffles and 1 PBSV for PacBio data, 1 Sniffles and 1 NanoSV for ONT data, and 1 Dovetail Hi-C) were merged using the same command for large SV integration from Survivor:

*Command: SURVIVOR merge $input.vcf.list 10000 1 0 0 0 10000 $output.vcf*

Small SVs (SV < 30kb and no translocations) from tumor-only calls from all samples and all tools (11 LongRanger for short deletions, 11 TNScope, 11 Novobreak, 11 Delly, 1 Sniffles and 1 PBSV for PacBio data, 1 Sniffles and 1 NanoSV for ONT data) VCF files were merged with the same command for small SV integration from Survivor as follows:

*SURVIVOR merge $input.vcf.list $window_size 1 0 0 0 $window_size $output.vcf*

In addition, SVs on ALT contigs or 10X Genomics’ blacklist regions were removed. For the high-confidence SV call set, we selected SVs that were represented in calls from at least 2 technologies and window size of 10kb for merging the SVs of the same type together.

#### Clustering analysis for merged SVs

The merged SVs are clustered based on SV type and SV sizes using a clustering algorithm to merge the SVs of the same type that require the two calls to have similar size (>50% similarity) and the two calls can be no more than the defined window size. We split the SVs into the separate files based on SV sizes and defined the clustering window size as follows:

- 50 to 100bp SV files use 50bp clustering window size
- 100 to 500bp SV files use 100bp clustering window size
- 500bp to 1kb SV files use 500bp clustering window size
- 1 to 30kb SV files use 1kb clustering window size
- 30kb above SVs or translocation event SV files use 10kb clustering window size

### Subsampling analysis

#### Illumina short-read data downsampling

Sequencing reads were downsampled using SAMtools version 1.6. A workflow was created in BGL called “Multi downsample BAM,” which ran the “SAMtools view” tool on all SAM or BAM files in a directory and includes an option to downsample the reads by a given fraction corresponding to the “-s” parameter in SAMtools view. The workflow indexed the resulting BAM files using “SAMtools index.” The workflow was used to generate all downsampled BAM files and index files and created a subset with defined read coverage.

BAM files from BWA [26] alignment of three replicated runs of WGS with 100X coverage on HCC1395 and HCC1395BL were merged using SAMtools [60] (version 1.8) for 200X or 300X coverages respectively. Newly created BAM files were then indexed and regrouped using Picard Tools [48] (version 2.17.11).

#### 10X Genomics data downsampling

Three replicates of the tumor sample which was sequenced at Novartis were selected for subsampling analysis. All three samples were merged using 10X Genomics LongRanger produced .mro files. The resulting bam file was subsampled to 100x using the bamtofastq tool provided by 10X Genomics. Each subsequent subsampling was generated from higher depth resulting in a bam file as input using the same subsampling method from 100x to 50x, 50x to 30x, 30x to 20x, and 20x to 10x.

#### Pacbio long-read data downsampling

The tumor mapped bam file mean coverage was ~39x. NGM-LR was used for the subsampling analysis. The bam file was subsampled to 30x, 20x, and 10x using the bbmp reformat.sh command.

*reformat.sh in=tumor.merge.ngmlr.dedup.bam out=pb.tumor.10x.bam mappedonly=t samplereadstarget=4172820 ref=hg38.fa -Xmx31g*

The SVs from each subsampling were split and merged using Survivor with the same method previously described for large and small SV integration based on window size. The merged SVs were taken as a union set from each call set rather than the filtered SVs.

### Annotation of high-confidence SVs

The annotation of the high-confidence SV call set used the AnnotSV software [29, 30]. AnnotSV furnishes breakpoint annotation to NGS including repeated sequences or G/C content. It starts by detecting the genomic overlaps between the input and the annotation features. It constructs an annotation based on the full-length SV and an annotation for each gene within the SV. Significant structure variants were compared with the Cancer Gene Census Project of the Catalogue Of Somatic Mutations In Cancer (COSMIC) database (GRCh38 · COSMIC v90) [61] to find variant genes are overlapping with the Cancer Gene Census list.

### Fusion gene analysis

We performed RNA-seq fusion gene analysis to further investigate fusion gene and translocation events in the genome. We used Star Fusion [62], Arriba [63] for fusion event detection. Each pipeline produces a set of fusion candidates. Gene fusion events found in the two tools were passed onto the next step as our high-quality fusion events. For tumor-specific fusion genes, the list of fusion genes that were also detected in normal samples was subtracted. The high-confidence genes from fusion genes were also compared with the high-confidence SV events to find overlap genes to produce the final integrated SV-Fusion gene set.

*Commands*:

*STAR --runThreadN 8 --genomeDir $ref_genome.fa.star.idx --readFilesIn $intput_R1_trimmed.fastq $inut_R2_trimmed.fastq --limitBAMsortRAM 31532137230 --outReadsUnmapped None --outSAMtype BAM SortedByCoordinate --alignIntronMax 200000 --alignMatesGapMax 200000 --alignSJDBoverhangMin 10 --alignSJstitchMismatchNmax 5 -1 5 5 --chimSegmentMin 12 --chimJunctionOverhangMin 12 --chimSegmentReadGapMax 3 --twopassMode Basic*

*STAR-Fusion --genome_lib_dir $ref_genome -J $Start_Chimeric.out.junction --output_dir Star_fusion_outdir*

*arriba -x Star_Fusion_Aligned.sortedByCoord.out.bam -c Star_Fusion_Chimeric.out.sam -g $ref_gencode.v24.annotation.gtf -a $ref_GRCh38.d1.dv1.fa -b $ref_blacklist_filter.bed -d $output_SV_for_arriva.tsv -o $output_arriba.tumor.with.sv.tsv*

### Reconstruction of cancer karyotypes for cancer cell line using RCK tool

HATCHet was used to generate allele-specific and clone-specific copy number values for matched tumor-normal samples using the recommended settings provided in the user documentation (http://compbio.cs.brown.edu/hatchet/). HATCHet-generated segment values were converted for processing with RCK using *rck-scnt-x2rck* following the author recommendations. Estimated copy number values within telomeric segments were extended from the closest estimated value reported by HATCHet. The high-confidence structural variants and converted HATCHet segment data were used as input for RCK. RCK was run using default parameters. RCK was run with the following command:

Rck –scnt segs_extend.tsv –acnt adj_X_removed.tsv

### Calculating SV calling frequency and select high-confidence call set

The frequency of a variation is defined by the ratio of a relative measure compared to the number of sample technical replicates tested which include library replicates and software replicates.

For both small and large SVs, the frequency of each SV was calculated on three levels: (1) per tool frequency, (2) per platform frequency, and (3) general consensus score.

Per tool frequency was calculated by counting the SVs detected by a software tool divided by the total count of replicates in each platform. For the 10X Genomics and Illumina data sets, the occurrence was divided by 11 (replicates). For PacBio and Hi-C samples, it was either 1 or 0 represent called or missing SVs by the software tool.

Per platform frequency was calculated by counting the SVs detected by the specific platform and divided by all replicates. For instance, the 10X Genomics platform-specific SV frequency is the count of the SVs detected by two software tools in 22 replicates. Since there was only one replicate and two software tools for PacBio data, the frequency of detection was either ½, 1, or 0. For Illumina, there were 44 technical replicates (11 from TNscope, 11 from Delly, 11 from Novobreak, and 11 from Manta). Therefore, the frequency was the count of the SVs detected by the above 3 software tools and divided by 44. The consensus score was assigned based on the total sum of per tool frequency for each SV. SVs which are called by at least two platforms were taken as high-confidence calls

## Supplementary Information

Additional file 1: Supplementary Table S1. WGS Data Sets from 5 NGS Technologies. WGS Libraries were made from the fresh DNA from HCC1395 and HCC1395BL cell lines. 42 Illumina-short read libraries were prepared by using Illumina TruSeq DNA PCR Free (1000ng) protocol and sequenced on 3 Illumina platforms including NovaSeq, Hiseq and HiSeq 10x across 6 sequencing centers. 22 10X Genomics WGS Linked-read libraries were prepared using 10X Chromium Genome V2 kit and sequenced on illumina HiSeq. 2 PacBio long-read protocol libraries were prepared with 10kb library protocol and sequenced on Sequel V2.1 chemistry. 2 Oxford Nanopore SQK-LSK109 ligation kit prepared libraries were run on MinONT flowcells. 6 Dovetail HiC libraries were prepared using the Dovetail SELVA Library Prep kit and sequenced on Illumina.

Additional file 2: Supplementary Table S2. Tool Specific SV Calls. SVs called by Novobreak, Manta, Delly and TNScope were using the tumor-normal pair mode to obtain tumor specific somatic calls, while the rest of the software called the SVs from tumor and normal samples separately. The tumor specific somatic calls were used Survivor software to subtract the SVs from matched normal sample.

Additional file 3: Supplementary Table S3. SV Initial Call Set. The initial callset were obtained from integration of tool specific SVs from multiple replicates using Survivor software, only the SVs present in at least two replicates were selected into the initial call set.

Additional file 4: Supplementary Table S4. SV High Confidence Consensus Call Set. The SVs from initial callset are further filtered based on consensus scores, SVs that were called in at least two platform call sets by multiple software tools were selected. Additional filtering applied to remove overlap SVs and SVs on the blacklist regions as well as the on three chromosomal regions with normal loss of heterozygosity LOH (chr6p, chr16q, and chrX) to obtain a high-confidence call set. multiple break point events were named as BNDs and further characterized in Additional file 15.

Additional file 5: Supplementary Table S5. SV Validation Call Set. A subset of SVs was selected based on consensus score for PCR validation. In addition, orthogonal methods: Bionano optical mapping, Affymetrix arrays, and RNA-seq were used to validate the SVs in the consensus call set.

Additional file 6: Supplementary Table S6. Bionano validated SVs. Bionano optical mapping was used to profile the SVs. The call set from Bionano were used to validate high confidence call set based on the method described in the methods section.

Additional file 7: Supplementary Table S7. PCR-based Validation. A subset of SVs from initial call set and high confidence call set were selected based consensus score for validation.

Additional file 8: Supplementary Table S8. Affymetrix Array Validation. The Affymetrix CytoScan Array was used for measuring copy number gain and loss.

Additional file 9: Supplementary Table S9. SVs Validated by RNA-seq. For fusion detection from RNA-seq data, we used Star Fusion and Arriba to produce a set of fusion candidates. Gene-fusion events found in the two tools were used to compare with the consensus SV call set to find overlap genes to produce the final integrated SV-Fusion gene call set.

Additional file 10: Fig. S1. Software Aligners and Caller Impact for SV Detection Sensitivity. Fig. S2. 10x Genomics Short Deletion Reproducibility. Fig. S3. Validation of the SV Consensus Call Set for HCC1395 Tumor Cell Line. Fig. S4. Translocation and Complex SV on Chromosome 17p of HCC1395 Cell Line. Fig. S5. Chromothripsis in HCC1395 Tumor Cell line. Fig. S6. Relative Sensitivity for SV Calls Across Platforms. Fig. S7. Translocation and Complex Events in HCC1395BL Cell Line. Fig. S8. Reproducibility of SVs in Consensus Call Set from 5 NGS Platforms and 11 Callers. Fig. S9. Examples of Complex Break Point Events (BNDs) Called by Different Technology and Software Tools.

Additional file 11: Supplementary Table S10. Pathogenic SVs. The annotation of the high confidence SV call set used the AnnotSV software to rank the pathogenic scores. We selected SVs annotated with score of 4 and 5 as well as SVs overlap the genes in Catalogue Of Somatic Mutations In Cancer (COSMIC) database.

Additional file 12: Supplementary Table S11. SVs Overlap Cancer Genes. High confidence consensus call set SVs were annotated using AnnotSV software. The significant structure variants were compared with the Cancer Gene Census Project of the COSMIC database (GRCh38 COSMIC v90) to find genes are overlapping with the Cancer Gene Census list.

Additional file 13: Supplementary Table S12. Tumor purity study. WGS Libraries were made from pooling the HCC1395 and HCC1395BL cell lines with various ratios (3:1, 1:1, 1:4, 1:9 and 1:19) to create mixtures. Libraries were prepared by using TruSeq DNA PCR Free (1000ng) protocol and sequenced on Illumina HiSeq 4000.

Additional file 14: Supplementary Table S13. a) Summary of large SVs with size > 30kb called from tumor purity titration and sequencing depth subsampling call sets. b) Summary of large SVs with size 50bp - 30kb called from tumor purity titration and sequencing depth subsampling call sets.

Additional file 15: Supplementary Table S14. Bionano somatic SVs called from tumor sample in at least two of the three replicates and were not normal sample.

Additional file 16: Supplementary Table S15. Summary of BND events characterized by multiple software tools from PacBio, ONT, HiC, Illumina, 10X and Bionano data sets.

Additional file 17. Review history

## Data Availability

All data files (FASTQ files, BAM file) are available on NCBI’s SRA database (SRP162370, https://www.ncbi.nlm.nih.gov/sra?term=SRP162370) [64]. The call set for structural variants in HCC1395, VCF files derived from individual WGS runs are available on NCBI’s ftp site (ftp://ftp-trace.ncbi.nlm.nih.gov/ReferenceSamples/seqc/Somatic_Mutation_WG ). Genomics DNA tested in the current study was prepared by ATCC using cell expansions from master banks of cells for the HCC1395 (ATCC, CRL-2324) and HCC1395BL (ATCC, CRL-2325) cell lines. gDNA aliquots from these preparations were distributed to the sequencing centers to perform WGS as described. For remaining gDNA aliquots, contact the corresponding authors. Contact ATCC for additional materials related to the HCC1395 and HCC1395BL cell lines.
