# Supplementary material for: Structural variant analysis of a cancer reference cell line sample using multiple sequencing technologies
Source: Genome Biol. 2022 Dec 13;23:255. doi: 10.1186/s13059-022-02816-6 (PMC9746098; doi:10.1186/s13059-022-02816-6)
Supplement: Supplementary file 10 — Additional file 10: Fig. S1. Software Aligners and Caller Impact for SV Detection Sensitivity. Fig. S2. 10x Genomics Short Deletion Reproducibility. Fig. S3. Validation of the SV Consensus Call Set for HCC1395 Tumor Cell Line. Fig. S4. Translocation and Complex SV on Chromosome 17p of HCC1395 Cell Line. Fig. S5. Chromothripsis in HCC1395 Tumor Cell line. Fig. S6. Relative Sensitivity for SV Calls Across Platforms. Fig. S7. Translocation and Complex Events in HCC1395BL Cell Line. Fig. S8. Reproducibility of SVs in Consensus Call Set from 5 NGS Platforms and 11 Callers. Fig. S9. Examples of Complex Break Point Events (BNDs) Called by Different Technology and Software Tools. [file 13059_2022_2816_MOESM10_ESM.pdf]

Additional file 10: Supplementary Information

Structural variant analysis of a cancer reference cell line sample using multiple sequencing technologies

Keyur Talsania[1,2#], Tsai-wei Shen[1,2#], Xiongfong Chen[1,2#], Erich Jaeger[3#], Zhipan Li[4#], Zhong Chen[5], Wangqiu Chen[5], Bao Tran[6], Rebecca Kusko[7], Limin Wang[8], Andy Wing Chun Pang[9], Zhaowei Yang[10], Sulbha Choudhari[1,2], Michael Colgan[11], Li Tai Fang[12], Andrew Carroll[13], Jyoti Shetty[6], Yuliya Kriga[6], Oksana German[6], Tatyana Smirnova[6], Tiantain Liu[5], Jing Li[10], Ben Kellman[9], Karl Hong[9], Alex Hastie[9], Aparna Natarajan[3], Ali Moshrefi[3], Anastasiya Granat[3], Tiffany Truong[3], Robin Bombardi[3], Veronnica Mankinen[14], Daoud Meerzaman[15], Christopher E. Mason[16], Jack Collins[1,2], Eric Stahlberg[2], Chunlin Xiao[17], Charles Wang[5]*, Wenming Xiao[11]*, Yongmei Zhao[1,2]*

[#]Contributed equally

*Correspondence should be addressed to: Yongmei.Zhao@nih.gov, Wenming.Xiao@fda.hhs.gov, and oxwang@gmail.com

Supplementary Figures

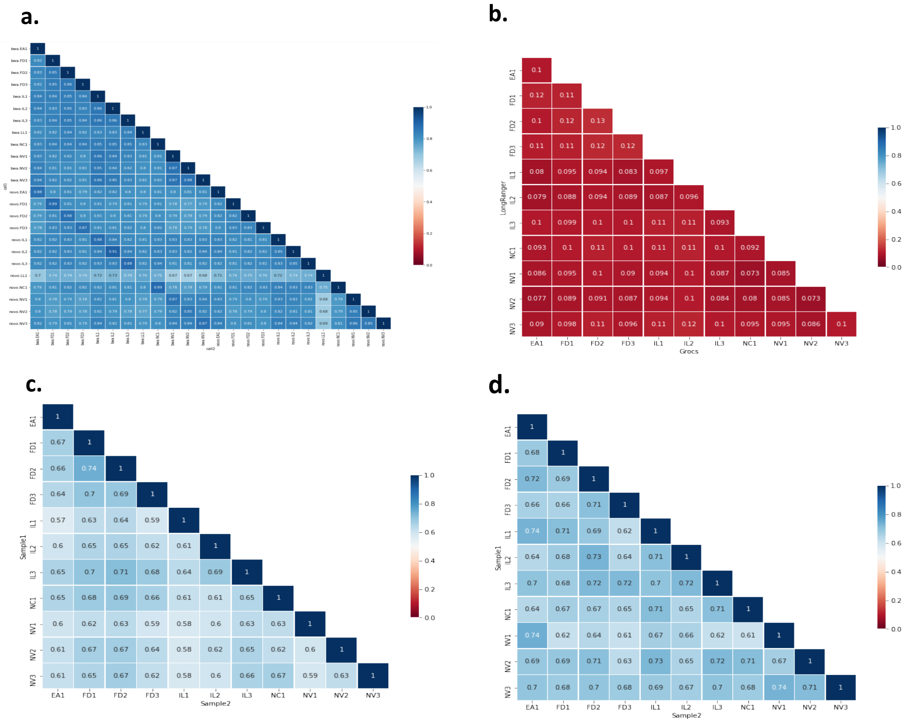

Fig. S1. Software Aligners and Caller Impact for SV Detection Sensitivity. (**a**) comparison of aligner effect. The matrix showed correlations between 24 pairs of replicates SV calling results which were generated by using BWA-mem or Novoalign for alignment and TNscope for SV calling. (**b**) comparison of variant caller effect between Longranger and Groc-SV. 11 pairs of 10x Chromium linked-read WGS replicates were compared. Observed low concordance calls between the two callers. (**c**) concordance among Longranger large SVs (>=30kbs) call results for 10x genomics data sets. The sequencing replicates were prepared by 5 different sequencing sites, same aligner (Lariat) and same caller (Long ranger) were used for data processing. (**d**) concordance among Groc-SVs results (large SVs includes DEL, INS, TRA, INV). The sequencing replicates were prepared by 5 sequencing sites, aligner (BWA-MEM) and caller (Groc-SVs) were used for mapping and variant calling.

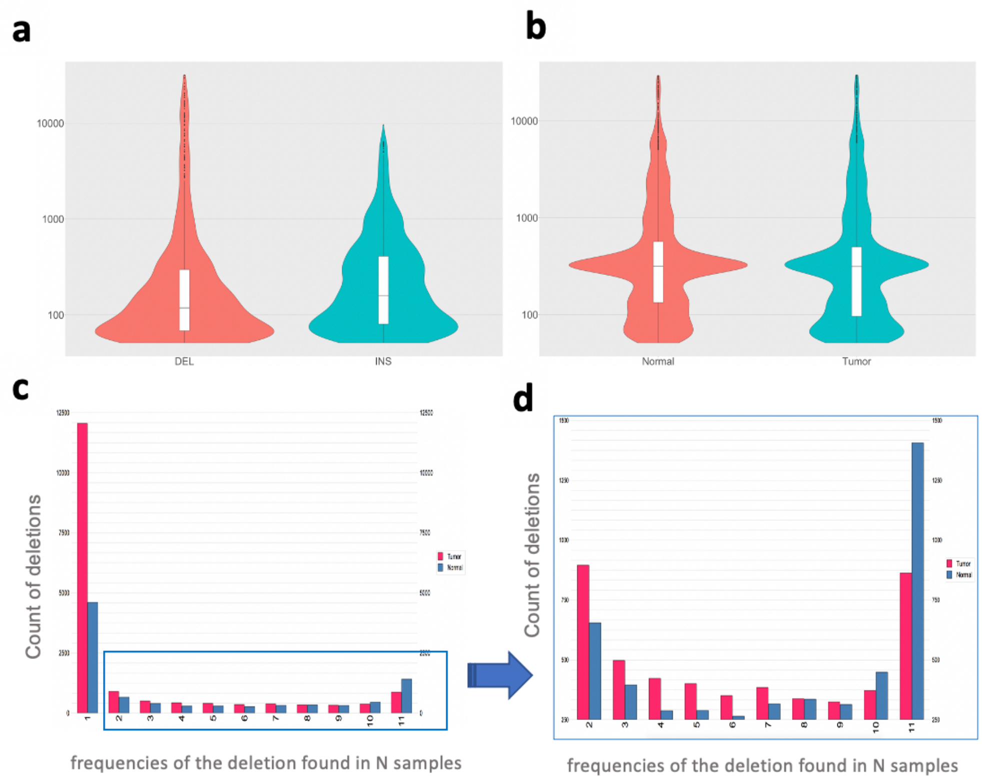

Fig. S2. **10x Genomics Short Deletion Reproducibility** (**a**) PacBio somatic call of deletions and insertions (**b**) 10X Genomics – short deletions size between 50kb – 30kbs. The Y axis for both plots denote the deletion sizes. High number of short deletions less than 1kb observed for 10x Long Ranger calls.**(c)** All short deletions with size between 50kb - 30kb called by 10x Long Ranger software (**d**) Short deletions called by 10X Long Ranger in at least two samples. The X axis denotes the frequencies of the deletion found in 1 sample, 2 sample, N sample. Y axis denotes the count number of short deletions in a particular frequency. Sample specify private calls are the highest frequency calls observed for 10x Long Ranger short deletions.

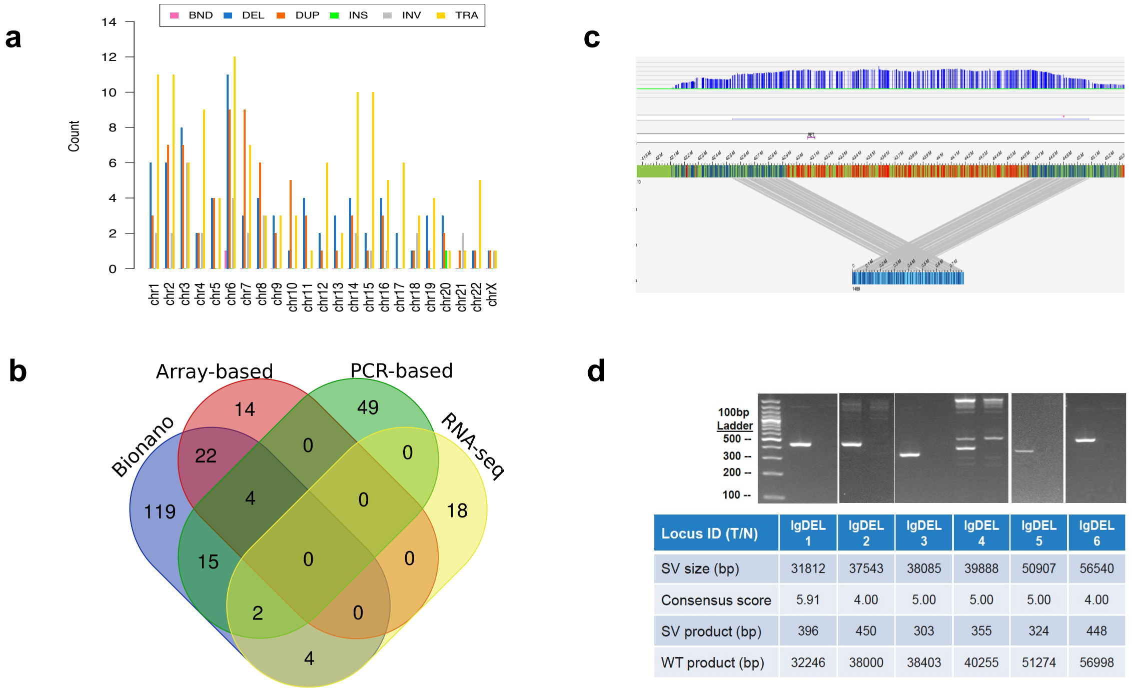

Locus ID (T/N)	IgDEL 1	IgDEL 2	IgDEL 3	IgDEL 4	IgDEL 5	IgDEL 6
SV size (bp)	31812	37543	38085	39888	50907	56540
Consensus score	5.91	4.00	5.00	5.00	5.00	4.00
SV product (bp)	396	450	303	355	324	448
WT product (bp)	32246	38000	38403	40255	51274	56998

Fig. S3. Validation of the SV Consensus Call Set for HCC1395 Cancer Cell Line. (**a**) Total number of SVs were validated by different methods for each SV type (**b**) SVs validated by each platforms and multiple NGS platforms. (**c**) one 2.5 Mbp amplification impacting *RET* gene was detected by multiple NGS platform including PacBio/Illumina and10x linked-read technologies at chr10: 42528,663 to chr10: 45.086,964 was confirmed by BioNano map on chromosome 10: 42,012,629-45,493,007, 3 - 6 copies; it is also confirmed by Affymetrix array at chr10:31813672 to chromosome 10: 45,092,199 with 3 copies gain. (**d**) PCR-based validation. The top showed the gel picture of the PCR product size bands and bottom showed the size of each SV and its corresponding PCR product size.

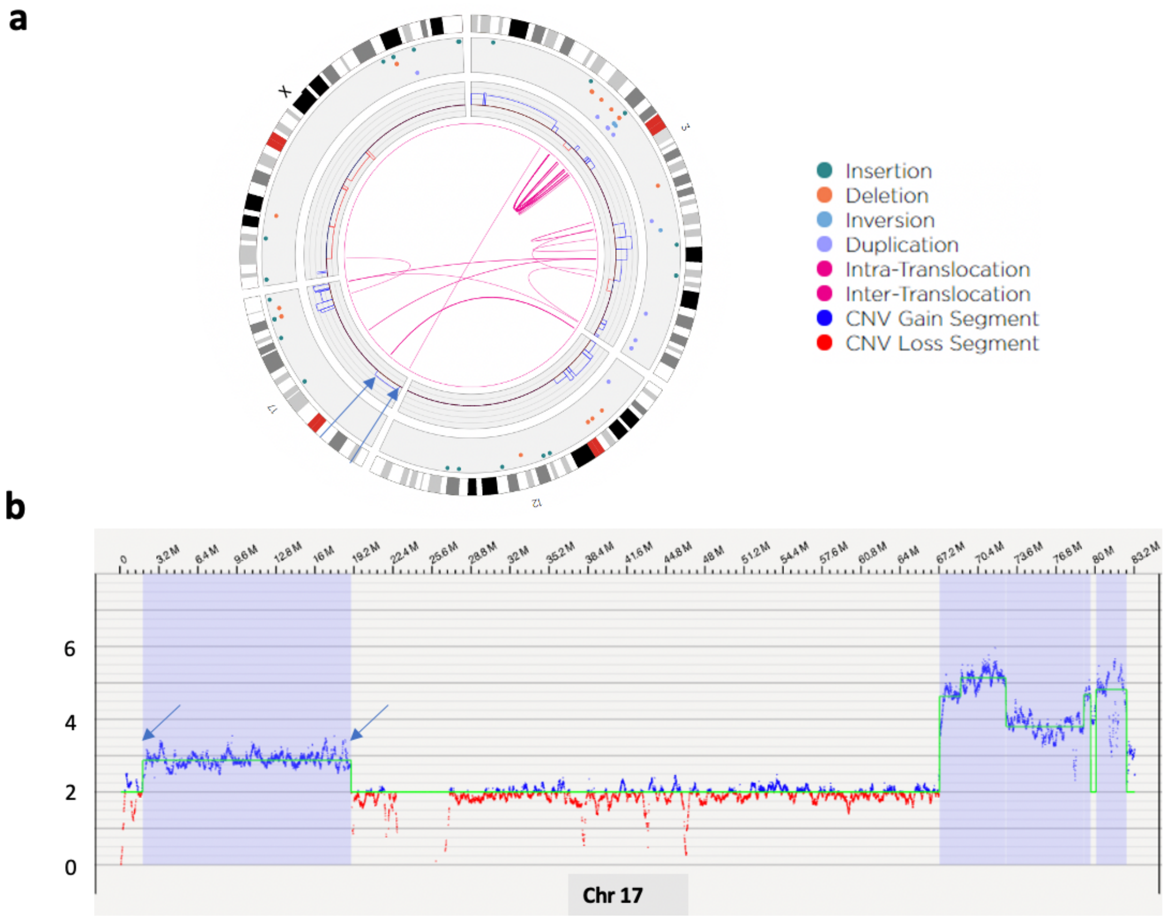

Fig. S4. Complex SV on Chromosome 17p of HCC1395 Cell Line. A 16.1 Mbp duplication on 17p (chr17:1,864,623-18,055,721) uniquely detected by Bionano genomics. This 3-copy duplication impacts multiple genes USP6, RABEP1, TP53, PER1, GAS7, MAP2K4, NCOR1 and FLCN. Furthermore, the duplication breakpoints coincide with t(3;17) and t(12;17). (**a**) The circos plot shows all the somatic variants on chr3, 12,17 and X. The breakpoints of the duplication of interest are indicated by the two blue arrows. (**b**) A view of the coverage profile of chromosome 17. The shaded regions are calls made by the CNV algorithm, and the one with two flanking blue arrows are the one 16.1 Mbp duplication.

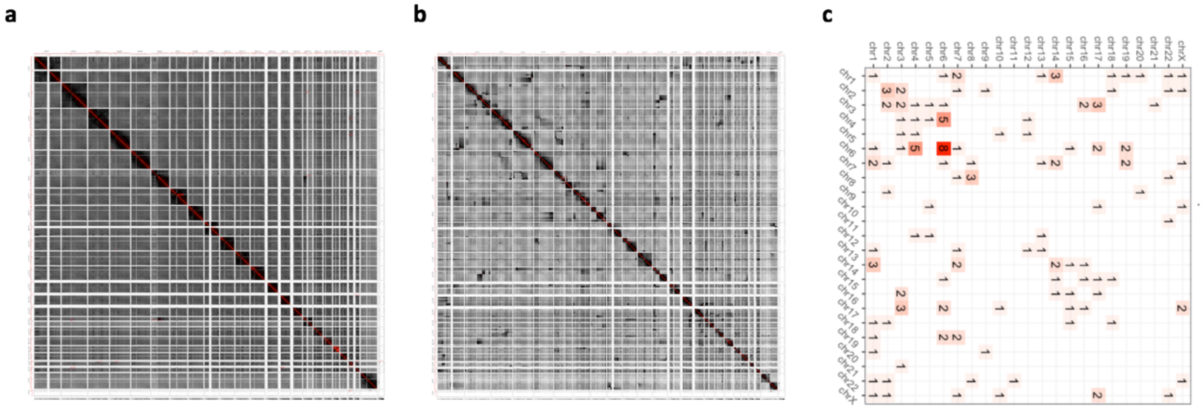

Fig. S5. Chromothripsis in the HCC1395 Tumor Cell line (a) Dovetail Selva generated Dot Plot for chromosomal view of HCC1395BL normal cell line. **(b**) Dovetail Selva generated Dot Plot for chromosomal view of HCC1395 tumor cell line. **(c**) Overview of the number of translocation events in HCC139 tumor cell line

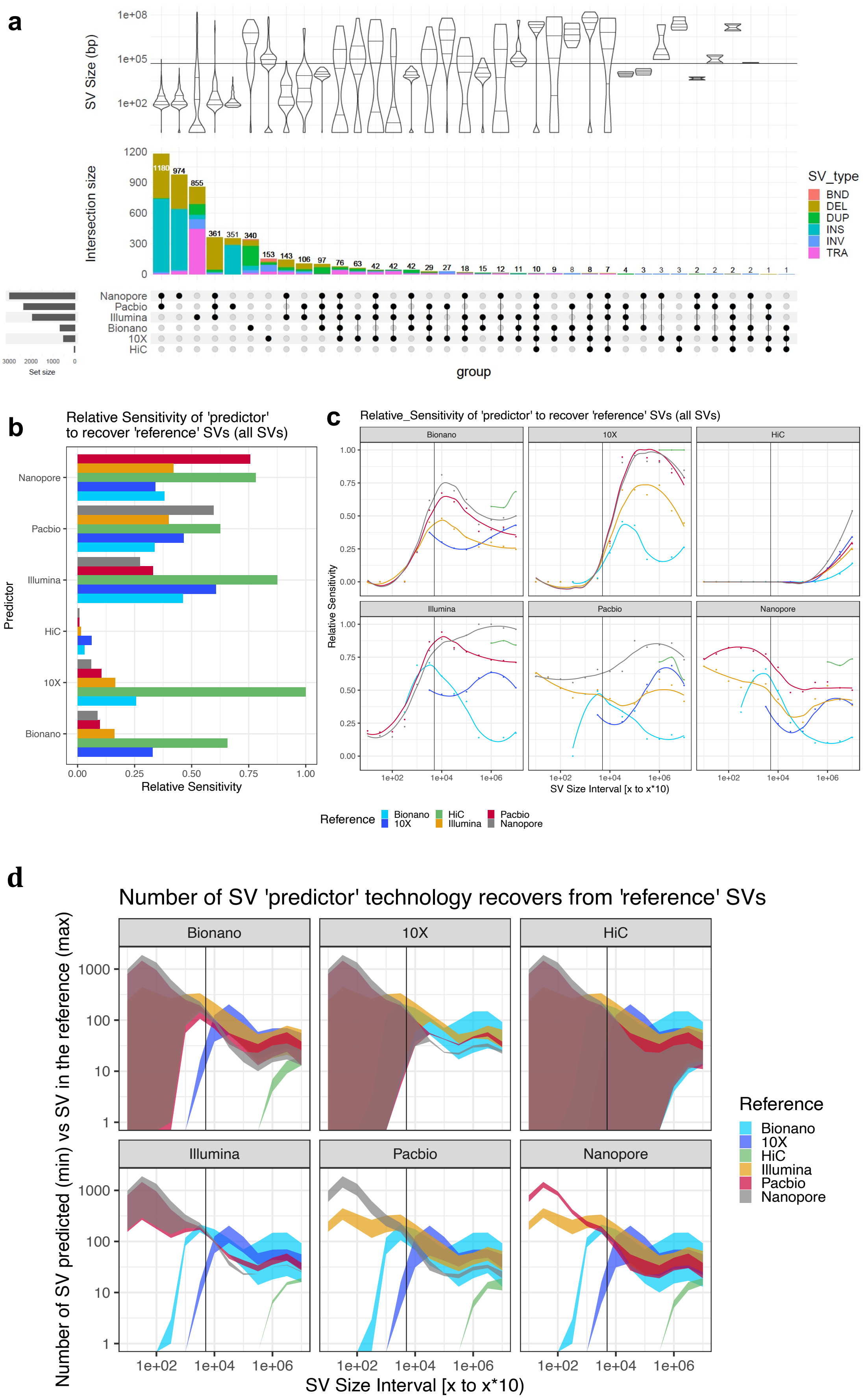
a
SV Size (bp)
1e+08
1e+05
1e+02
Intersection size
1200
900
600
300
0
1180
974
855
361
351
340
153
143
106
97
76
63
42
42
42
29
27
18
15
12
11
10
9
8
8
7
4
3
3
3
2
2
2
2
1
1
SV_type
BND
DEL
DUP
INS
INV
TRA
Nanopore
Pacbio
Illumina
Bionano
10X
HiC
3000
2000
1000
0
Set size
group
b
Relative Sensitivity of 'predictor'
to recover 'reference' SVs (all SVs)
Predictor
Nanopore
Pacbio
Illumina
HiC
10X
Bionano
0.00
0.25
0.50
0.75
1.00
Relative Sensitivity
c
Relative_Sensitivity of 'predictor' to recover 'reference' SVs (all SVs)
Bionano
10X
HiC
Illumina
Pacbio
Nanopore
Relative Sensitivity
1.00
0.75
0.50
0.25
0.00
1e+02
1e+04
1e+06
SV Size Interval [x to x*10)
Reference
Bionano
10X
HiC
Illumina
Pacbio
Nanopore
d
Number of SV 'predictor' technology recovers from 'reference' SVs
Bionano
10X
HiC
Illumina
Pacbio
Nanopore
Number of SV predicted (min) vs SV in the reference (max)
1000
100
10
1
1e+02
1e+04
1e+06
SV Size Interval [x to x*10)
Reference
Bionano
10X
HiC
Illumina
Pacbio
Nanopore

Fig. S6. Relative Sensitivity for SV Calls Across Platforms. (a) Comparison of SVs called by different platform including Nanopore, PacBio, Illumina short-read, Bionano, 10X Chromium linked-read, and HiC. Upset plot expanding on data similarities highlighted in **Figure 2a**. Intersection size is split into SV-type including multiple breakpoints SV events (BND), deletions (DEL), duplications (DUP), insertions (INS), inversions (INV), and inter-chromosomal translocations (TRA). Violin plots indicate the distribution of SV-sizes, in base-pairs (bp), within the vertically aligned with overlap-sets; horizontal lines within the violin plots indicate the 1st quantile, median, and 3rd quantile. A horizontal line is included in the violin plots to indicate 100kb size. **(b)** Relative sensitivity was calculated for all SV calls. **(c)** Relative sensitivity was calculated for all SV calls and stratified by size, between x and x*10 base pairs (x-axis) where x is the vertically aligned value on the x-axis. Relative sensitivity (x-axis in **b,** y-axis in **c**) is the proportion of all SV calls include SVs from initial call set defined in **Additional file 3: Table S3** and Bionano call set from **Additional file 15: Table S14** called by a reference platform (color) recoverable using another technology (y-axis in **b**, sub-panels in **c**) across different platforms. **(b)** Relative sensitivity of "predictor' to recover 'reference' SVs. Ribbon plot expansion on relative sensitivity to show the numerator (smallest shaded y-coordinate; number of reference SVs (color) found by the predictor platform (subpanel)) and the denominator (largest shaded y-coordinate; number of reference SVs (color)); the numerator at each size interval is the smallest vertically aligned y-coordinate within the shaded region while the denominator is the largest vertically aligned y-coordinate.

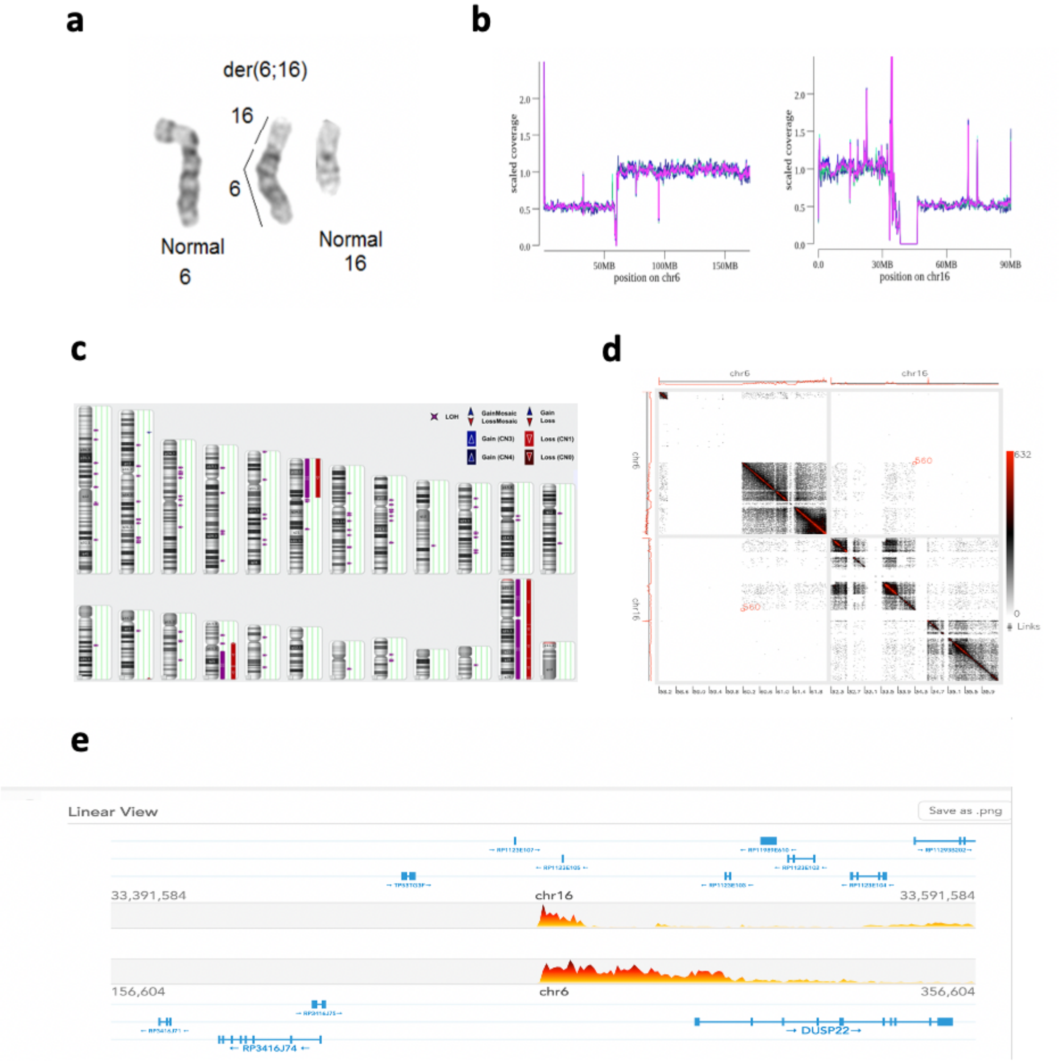

Fig. S7. Translocation and Complex Events in HCC1395 BL Cell Line. (**a**) Karyotyping showed fusion events of Chromosome 6 (chr6 p22) and Chromosome 16 (chr16 q21). (**b**) Global whole genome read-depth based analysis from all 3 WGS data sets (Illumina, PacBio and 10x Genomics) showed copy number change on Chr 6 p22 and Chr16 q21 (**c**) CytoScan® HD Assay confirmed HCC1395 translocation complex events occurred between Chr 6 p22 and Chr16 q21. (**d**) HiC detected a complex translocation event at chr6:60,243,000 and chr16:34,260,000. (**e**) 10x Genomics technology detected a 100kb translocation event between chr6:257,000 and chr16:32,280,000.

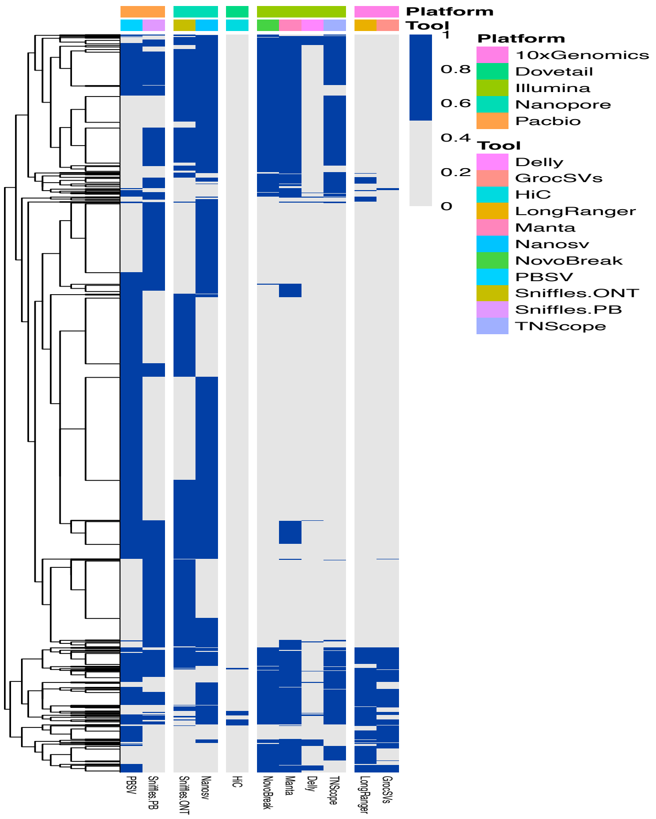

Fig. S8. Reproducibility of SVs in Consensus Call Set from 5 NGS Platforms and 11 Callers. Heatmap was generated based on SV location on genome and SV frequency which was calculated based on the method section of Calculating SV Calling Frequency and Consensus Call Set. Software tools displayed in the plots are: GrocSVs, Long Ranger, TNscope, Delly, Manta, Novobreak, HiC (Selva), Novasv, Sniffles.ONT for Nanopore data, Sniffles.PB for PacBio data, PBSV. The heatmap color denotes the SV frequencies detected by each tool for a technology, the dendrograms along the side of the heatmap show similarity and variability how the SVs are clustered.

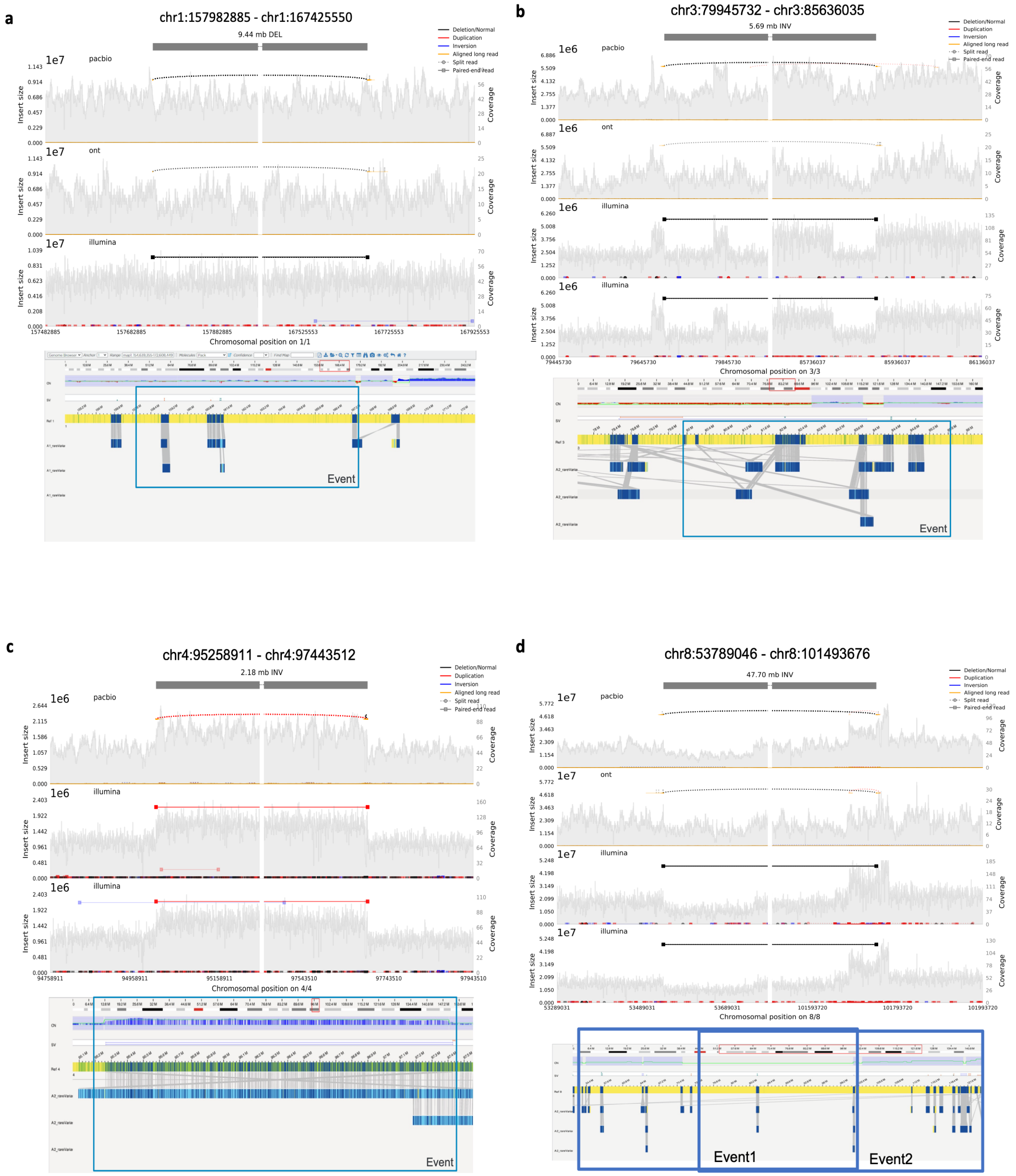
a
chr1:157982885 - chr1:167425550
9.44 mb DEL
Deletion/Normal
Duplication
Inversion
Aligned long read
Split read
Paired-end read
pacbio
ont
illumina
Insert size
Coverage
Chromosomal position on 1/1
Event
b
chr3:79945732 - chr3:85636035
5.69 mb INV
Chromosomal position on 3/3
Event
c
chr4:95258911 - chr4:97443512
2.18 mb INV
Chromosomal position on 4/4
Event
d
chr8:53789046 - chr8:101493676
47.70 mb INV
Chromosomal position on 8/8
Event1
Event2

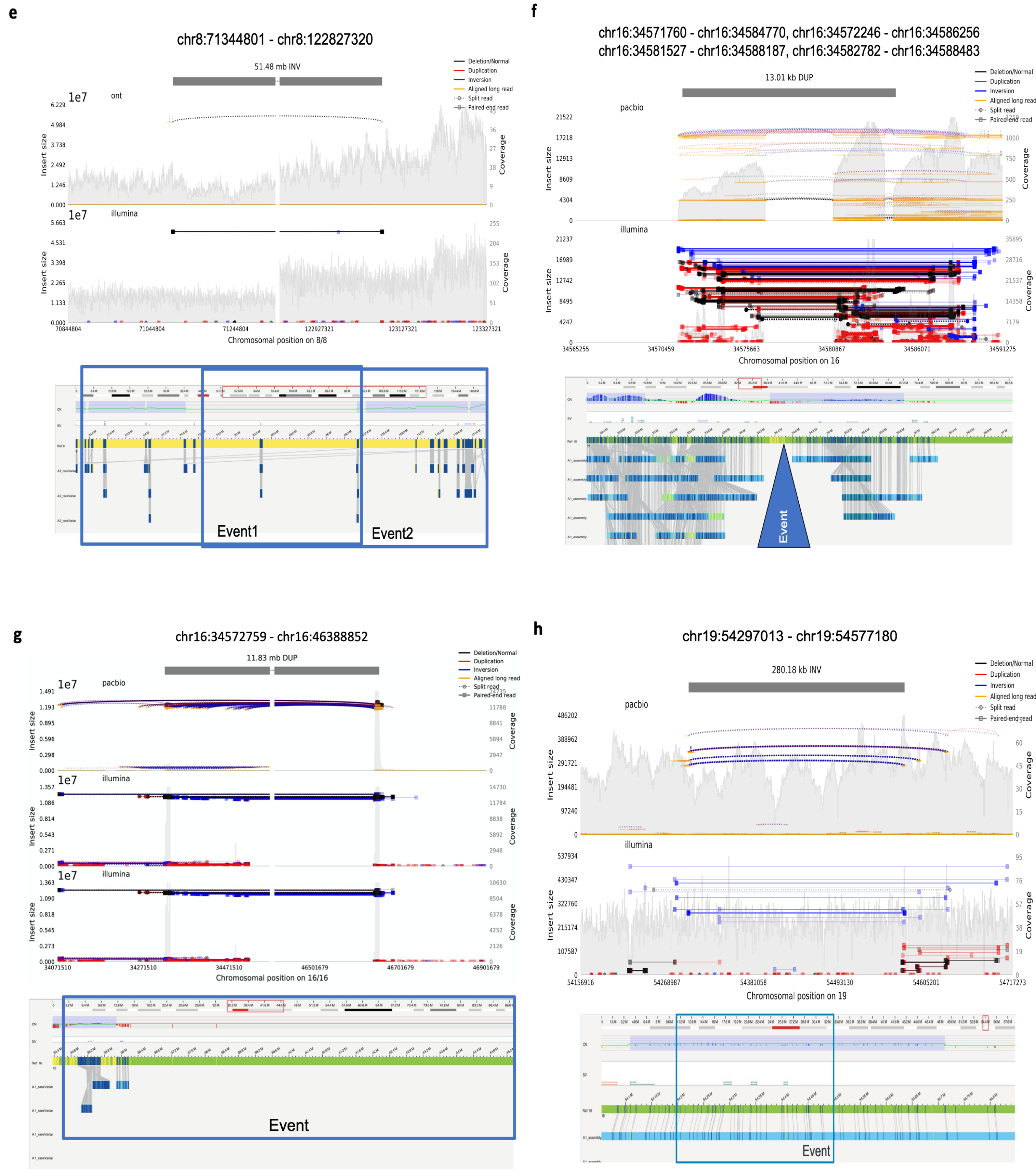

Fig. S9. Examples of Complex Break Point Events (BNDs) Called by Different Technology and Software Tools. Plots were generated by using SamPlot tool to show multiple break point events in regions displayed in each panel. The genome read coverage and structural variant events were profiled from PacBio, Nanopore (ONT), Illumina, and Bionano data. The summary of each BND event characterization was described in **Additional File 16: Table S15**.
