# Supplementary material for: Structural variant analysis of a cancer reference cell line sample using multiple sequencing technologies
Source: Genome Biol. 2022 Dec 13;23:255. doi: 10.1186/s13059-022-02816-6 (PMC9746098; doi:10.1186/s13059-022-02816-6)
Supplement: Supplementary file 17 — Additional file 17. Review history [file 13059_2022_2816_MOESM17_ESM.docx]

Review History

**First round of review**

**Reviewer 1**

**Were you able to assess all statistics in the manuscript, including the appropriateness of statistical tests used?** Yes, and I have assessed the statistics in my report.

**Comments to author:**

The authors aimed to evaluate somatic structural variation analysis. Multiple sequencing technologies were applied to generate data for matched normal-tumor cell lines, HCC1395 and HCC1395BL. In general, a lot of work have been done to combine and compare technologies to generate a high-confidence consensus SV call set. This work would be of great interest to both technology development and clinical application, if done properly. Here are a few comments.

Major:

1. As the authors also mentioned that primary tumor samples are heterogenous, on which variant calling is substantionally more challenging than what is presented here on cell lines.

Although the authors examined tumor purity by spiking in tumor DNAs into normal ones, those samples are still not as complex as real tumor ones. Thus I would recommend using "a cancer reference cell line sample" rather than "a cancer reference sample".

2. Survivor was applied to merge vcf files from multiple callers. It is fine to do so on cell lines but I doubt this approach would perform well on real tumor samples due to tumor heterogeneity. Besides, SVs were merged using multiple window sizes depending on the size of the events. I wonder why SV calls were divided into 5 groups with different merging parameter sets. The authors did not evaluate the breakpoint precision of the raw calls and the selection of paremeters need justification. How those parameters impact final merging result?

3. PacBio HiFi technology has demonstrated its power in assembling complex regions of the genome and led to much better SV calling. It would be ideal to include HiFi data in this study.

Minor:

1. In "Materials and methods" file, there have some mis-ordered sentences or paragraphs. E.g. The commend for TNscope (line 292 to line 295) should follow TNscope description (line 283 to line 285) rather than Manta description (line 288 to line 289). In addition, the bioinformatics analysis of Nanopore sequencing data is missed from "Materials and methods" file.

2. What is the percentage of final high-confidence consensus call set was detected from Oxford Nanopore?

3. For the event with mutiple breakpoints, it is necessary to characterize the components of each event.

4. The authors applied 4 independent validation methods to evaluate the call set while the false positive rate was not provided. It would be great if FDR would be provided when possible.

5. Line 82, not all long-read technologies have a high sequencing error rate as 10-15%. Please update.

6. Line 71 "challengingto"

**Reviewer 2**

**Were you able to assess all statistics in the manuscript, including the appropriateness of statistical tests used?** Yes, and I have assessed the statistics in my report.

**Comments to author:**

The manuscript of Talasania et al, described the efforts of the SEQQC2 consortium to establish a reference structural variation truth set for a pair of tumor/normal cell lines by applying multiple sequencing technologies and harmonizing their outputs. This is a companion manuscript to their marker paper on the project, here focused on the challenging problem of structural variants identification (SV). In a sense this project mirrors efforts by the Genome-in-a-Bottle (GiaB) consortium with their reference materials and truth sets for the genomes of selected cell lines widely consented for research and commercial use. The main contribution of this work is the focus on somatic structural variants, as one of the cell lines analyzed in this work is derived from a Breast cancer tumor and thus includes more complex SVs that are rarely observed in normal tissues. The reference data generated in this work thus provides a tool to benchmark new sequencing technology aiming to decode SVs in cancer cells and to aid in the development of new analysis algorithms to analyze data from tumor tissues by different sequencing technologies. Importantly, the cell lines analyzed here are widely available through a commercial provider (ATTC), and thus can be used widely by any party for such endeavors. The only drawback is that the cell lines may not be fully consented for commercial applications like in GiaB, potentially limiting some applications such as producing derivatives commercial products. Nonetheless, this work provides a very useful dataset that can be readily used for the goals described above.

The manuscript is well written and the methods and supplementary materials provide ample details to understand the derivation of the datasets. The authors provide a reference consensus call set data to be used as reference truth set for the cell lines, including specifically the list of somatic variants, and also the intermediate datasets used for deriving the final consensus call set (at the associated ftp site). The authors devised a validation strategy which is quite appropriate and provides good evidence of the quality of the consensus call set. While the authors made some specific analysis decisions that may be not as common (e.g. the use of some specific variant calling algorithms versus others), I think the combinations of tools is reasonable to provide a high quality set of calls.

While this manuscript is very good, and I believe this report should be published promptly, I provide some comments below.

1. Using consensus calling across multiple callers for a type of SV reduced false positive rate at the expense of sensitivity. This strategy will certainly provide a very good call set, but one wonders what are we missing. Can you provide an estimate of the false negative rate of the consensus call set?

2. The results about the impact of mixtures between normal and tumor cell lines is somehow embedded in the discussion section, while I think this is a very important results and merits its own section and figures rather than in supplemental materials. This analysis is very relevant to those trying to design assays to sequence tumor tissue with variable rates of purity. I would suggest a figure that plots the sensitivity across different rates of mixtures, with lines representing different types of variants and at various depths of coverages (may be separate panels for different depths) to make very clear the impact of mixture levels vs depth of coverage, quite useful for practical purposes. This could be a figure in the main body of the paper rather than just supplemental tables instead of supplemental tables. Additional supplemental figures could also provide similar analysis for different platforms. I think this information would be one of the major learnings of this work.

3. Figure 2's legend appears to have some errors. References to the panels d to g appear to be mislabeled.

4. Can you describe in more detail how you benchmarked the comparisons between the consensus reference call set and a given technology call set. Benchmarking a reference set of SV calls to a second call set is not a trivial problem and the methods used to perform this function need to be described and validated.

**Authors Response**

**Point-by-point responses to the reviewers’ comments:**

**Reviewer #1:** The authors aimed to evaluate somatic structural variation analysis. Multiple sequencing technologies were applied to generate data for matched normal-tumor cell lines, HCC1395 and HCC1395BL. In general, a lot of work have been done to combine and compare technologies to generate a high-confidence consensus SV call set. This work would be of great interest to both technology development and clinical application, if done properly. Here are a few comments.

Major:

   1. As the authors also mentioned that primary tumor samples are heterogenous, on which variant calling is substantially more challenging than what is presented here on cell lines.

Although the authors examined tumor purity by spiking in tumor DNAs into normal ones, those samples are still not as complex as real tumor ones. Thus I would recommend using "a cancer reference cell line sample" rather than "a cancer reference sample".

**RESPONSE:** We would like to thank the reviewer for the insightful suggestion. Yes, we fully agree with reviewer that using “a cancer reference cell line sample” rather than “a cancer reference sample” is more precise. We have updated the manuscript to address this point.

2. Survivor was applied to merge vcf files from multiple callers. It is fine to do so on cell lines but I doubt this approach would perform well on real tumor samples due to tumor heterogeneity. Besides, SVs were merged using multiple window sizes depending on the size of the events. I wonder why SV calls were divided into 5 groups with different merging parameter sets. The authors did not evaluate the breakpoint precision of the raw calls and the selection of parameters need justification. How those parameters impact final merging result?

**RESPONSE:** We thank the reviewer for the critical comment regarding the Survivor algorithm limitation for handling tumor SV events due to tumor heterogeneity.

Previous work used the Survivor algorithm to merge SVs from multiple call sets with a 1kb size window (Jeffares, D. *et al. Nat Commun* **8,** 14061 2017). This study showed that by selecting SVs called by at least two different variant callers from a high-quality SV consensus call set can significantly reduce false-positive rates.

In our study, the initial 82 raw call sets were generated from multiple algorithms and platforms using multiple replicates of the same cell line, we expect there are large degrees of redundancy. We applied a systematic approach for duplicate SV detection and removal. For initial consensus call set integration, we used 5 breakpoint window sizes rather than 1kb window size in order keep precise breakpoints called by SV callers for smaller SVs between 50bps – 1kb sizes. SV events such as insertions, deletions, intra-chromosomal inversions, inter-chromosomal translocations, and complex breakpoint events were called in a tool-specific manner. We calculated the consensus scores based on Survivor merged call set and removed the SVs that were only found in one of the 82 initial raw call sets. In addition, we filtered SVs overlapping the blacklisted regions of the reference genome.

For the high confidence call set, we took a more stringent protocol and performed two additional passes for removing duplicate calls. The consensus calls from multiple samples and tools require at least two technologies from two software tools to agree on the same variant based on SV types and SV sizes. In addition, we applied a clustering approach to further merge SVs of the same type if the SVs had a reciprocal overlap >50%. By using this clustering approach, we removed 79 additional SVs that were missed by Survivor merge approach. The 79 SVs includes 1 deletion, 1 duplication, 1 Insertion, 27 reciprocal Inversions and 49 reciprocal translocation events. The results of filtered consensus calls are provided in the supplementary table 3 & 4.

3. PacBio HiFi technology has demonstrated its power in assembling complex regions of the genome and led to much better SV calling. It would be ideal to include HiFi data in this study.

**RESPONSE:** We completely agree with the reviewer that with the high accuracy (>99.9%) and long read length (up to 25kb) achieved by PacBio HiFi technology, it would provide a better view of SVs in difficult genomic regions with short tandem repeats (STR), high G/C content, telomeres, centromeres, and highly repetitive sequences. It may also provide better resolution for SV breakpoints and junctions. However, due to the resource limitation and time constrain of the study, we won’t be able to include the HiFi data in this study.

Minor:

1.  In "Materials and methods" file, there have some mis-ordered sentences or paragraphs. E.g. The commend for TNscope (line 292 to line 295) should follow TNscope description (line 283 to line 285) rather than Manta description (line 288 to line 289). In addition, the bioinformatics analysis of Nanopore sequencing data is missed from "Materials and methods" file.

**RESPONSE:** Thank you for catching up the mis-ordering of the method for TNScope and Manta description. We have corrected the paragraph in the “Materials and Methods” file. We also added the bioinformatics analysis of Nanopore sequencing data method to the “Material and Methods” under the “Oxford Nanopore Sequencing Data SV Analysis” section.

2.  What is the percentage of final high-confidence consensus call set was detected from Oxford Nanopore?

**RESPONSE:** In our high-confidence consensus call set, Illumina, PacBio, Nanopore, 10X Genomics (large SVs >=20kb only) and Hi-C (large SVs >=1 Mb) have each detected 47%, 86%, 87%, 84% and 2% of the final high-confidence call set respectively.

Among all the five platforms, we observed that long-read based technologies such as PacBio, Nanopore and 10X Genomics linked-reads detected a high number of SVs that are missed by short-read based technologies. In addition, SV call sets detected by PacBio and Nanopore have the highest overlap compared to any other platform’s (86% for PacBio and 87% for Nanopore detected SVs in the high confidence call set.

We expanded the cross-platform comparison analysis by using the initial consensus call set to assess the relative sensitivity for SV calling across platforms. The results were included as a new section “Assessment of Relative Sensitivity for SV Calls across Platform” for the revised manuscript.

3.  For the event with multiple breakpoints, it is necessary to characterize the components of each event.

**RESPONSE:** Thank you, this is an insightful point. We extracted the 11 multiple breakpoint events (BNDs) from high confidence call set to characterize each event called by 11 NGS SV callers and Bionano caller. In addition, we visualized the SV regions using SamPlot (Belyeu, J.R. *et al*. *Genome Biol.*  2021) and attempt to uncover the associated complexity. However, due to the current software algorithm limitation, some of the complex events were not fully characterized. The following plot shows multiple events in region chr3:79,945,732 - chr3:85,636,035. The long-read callers identified inversion and deletion events from PacBio and Nanopore (ONT) data. Short-read callers identified deletions from Illumina data, 10x linked-read callers identified inversion events. Based on Bionano data, several events were identified including copy number gain, 3 insertions, 1 duplication, several inversions across the region.

**Extra figure for Reviewer only***:* Plot generated by SamPlot tool to show BND event in region chr3:79,945,732 - chr3:85,636,035. The genome read coverage and structural variant events were profiled from PacBio, Nanopore (ONT), Illumina, and Bionano data. The plot shows inversion and deletions were called from PacBio, inversion was identified from ONT data, deletions were called from Illumina data. Bionano data showed several events including copy number gain, 3 insertions, 1 duplication, several inversions across the region.

We observed similar patterns for other BND events, several SVs adjacent or overlap with each other in the same locus. This reveals the cancer genome heterogeneity. Detecting low allele frequency structural variants in subclonal tumor cell populations remains challenging due to sequencing technology and software algorithm limitations. We hope those complex SV events will be further characterized in future studies as long-read technologies continuously increase read-length limits and new SV algorithms are developed for improved sensitivity and accuracy.

A summary of the multiple breakpoint events is shown in the following table. The additional plots for visualizing the BND events were included as “Extra_data_file_for_reviewers_only” in the submission.

| **SV_id** | **BND Complex Event Summary** |
| --- | --- |
| chr1_157982885_chr1_167425550_+- | Pacbio, ONT and Illumina show a small deletion within a large deletion. From Bionano data, it shows the BND event occurs within a large copy number gain. The event contains 2 insertions (green), 2 deletions (orange) and flanked by 1 inversion. |
| chr3_79945732_chr3_85636035_+- | PacBio data callers called events as inversion and deletions, Nanopore caller called inversion event. Illumina short-read callers called deletions, 10x Longranger and Groc-SVs called inversion. Bionano showed several events including copy number gain, 3 insertions, 1 duplication, several inversions across the region. |
| chr4_95258911_chr4_97443512_++ | Based on Illumina, PacBio and Nanopore data, the event is characterized as a large copy number gain and an inversion flanking the region. |
| chr8_53789046_chr8_101493676_+- | PacBio data shows deletion. Illumina data shows duplication and a large deletion. Bionano data shows copy number gains, insertions, deletions. The event contains an inversion, copy number gains/duplications, and a large deletion flanking the deletion region. |
| chr8_71344801_chr8_122827320_+- | Ont data detected an inversion and duplication. Illumina data showed deletion, duplication and inversion events in the region. Bionano shows copy number gains, insertions, deletions. The event contains an inversion, large deletion, and copy number gain events flanking the deletion region |
| chr16_34571760_chr16_34584770_+- | PacBio and Illumina data showed duplication and deletions, or inversions however, Bionano data showed there is no events called in the region, but the event flank regions have large copy number gains, inversions and deletion events called by Bionano Access software. |
| chr16_34572246_chr16_34586256_-+ | PacBio and Illumina data showed duplication and deletions, or inversions however, Bionano data showed there is no events called in the region, but the event flank regions have large copy number gains, inversions and deletion events called by Bionano Access software. |
| chr16_34581527_chr16_34588187_+- | PacBio and Illumina data showed duplication and deletions, or inversions however, Bionano data showed there is no events called in the region, but the event flank regions have large copy number gains, inversions and deletion events called by Bionano Access software. |
| chr16_34582782_chr16_34588483_+- | PacBio and Illumina data showed duplication and deletions, or inversions however, Bionano data showed there is no events called in the region, but the event flank regions have large copy number gains, inversions and deletion events called by Bionano Access software. |
| chr16_34572759_chr16_46388852_+- | Pacbio data shows one large duplication event in the region. Illumina data showed both duplication and inversion. Bionano data shows one duplication, inversion, one insertion and flanking a centromere. The event was characterized as duplication, inversion and flanking the centromere region. |
| chr19_54297013_chr19_54577180_++ | Pacbio data shows inversion events were called, while Illumina data shows both inversion and duplication events. Bionano data shows a large copy number gain with 3 insertions inside the region. The SV is characterized as inversion and duplication |

4.  The authors applied 4 independent validation methods to evaluate the call set while the false positive rate was not provided. It would be great if FDR would be provided when possible.

**RESPONSE:** Thank you for the comment. We chose a total of 110 SV events from different size ranges including DEL, DUP, INV, and TRA from the initial call set for PCR validation. The validation rates for the DEL, DUP, INV, and TRA were 93%, 75%, 54% and 25%, respectively. However, incapable detection of one SV in our call set by a chosen validation method does NOT necessarily mean a true false positive call. For example, a non-detection call made by PCR validation might be simply due to a poor PCR primer set designed, whereas a non-confirmation by Cytogenetic Array technology might be due to its limitation on the resolution and detection range. Therefore, for the 4 independent validation methods applied in this study, we could not conclude if it is a false negative of validation method or a false positive of our SV calling. As a result, we cannot provide FDR on our SV reference call set with four validation methods

As demonstrated in our study as well as by other studies such as Dixon, J.R. *et al.* *Nat Genet.* 2018, to date, there is still no universal method that can provide an accurate detection for the spectrum of SV type and size. We believe that an integrative approach will allow a comprehensively identification of SVs in the cancer genome, which is our recommended method for cancer genome SV detection.

5. Line 82, not all long-read technologies have a high sequencing error rate as 10-15%. Please update.

**RESPONSE:** We are fully aligned with the reviewer’s suggestion and we have updated the sentence as “Long-read technologies can easily span break points but have a high sequencing error rate from raw data (~10-15). Although the latest chemistry from the long-read technologies such as PacBio HiFi reads achieve greater accuracy (> 99.9%) from consensus reads”

 6. Line 71 "challengingto"

**RESPONSE:** We thank the reviewer for catching up the typo. We have now corrected the word as “challenging to” on line 74 of revised manuscript.

**Reviewer #2:** The manuscript of Talasania et al, described the efforts of the SEQQC2 consortium to establish a reference structural variation truth set for a pair of tumor/normal cell lines by applying multiple sequencing technologies and harmonizing their outputs. This is a companion manuscript to their marker paper on the project, here focused on the challenging problem of structural variants identification (SV). In a sense this project mirrors efforts by the Genome-in-a-Bottle (GiaB) consortium with their reference materials and truth sets for the genomes of selected cell lines widely consented for research and commercial use. The main contribution of this work is the focus on somatic structural variants, as one of the cell lines analyzed in this work is derived from a Breast cancer tumor and thus includes more complex SVs that are rarely observed in normal tissues.  The reference data generated in this work thus provides a tool to benchmark new sequencing technology

    aiming to decode SVs in cancer cells and to aid in the development of new analysis algorithms to analyze data from tumor tissues by different sequencing technologies. Importantly, the cell lines analyzed here are widely available through a commercial provider (ATTC), and thus can be used widely by any party for such endeavors. The only drawback is that the cell lines may not be fully consented for commercial applications like in GiaB, potentially limiting some applications such as producing derivatives commercial products. Nonetheless, this work provides a very useful dataset that can be readily used for the goals described above.

    The manuscript is well written and the methods and supplementary materials provide ample details to understand the derivation of the datasets. The authors provide a reference consensus call set data to be used as reference truth set for the cell lines, including specifically the list of somatic variants, and also the intermediate datasets used for deriving the final consensus call set (at the associated ftp site). The authors devised a validation strategy which is quite appropriate and provides good evidence of the quality of the consensus call set. While the authors made some specific analysis decisions that may be not as common (e.g. the use of some specific variant calling algorithms versus others), I think the combinations of tools is reasonable to provide a high quality set of calls.

While this manuscript is very good, and I believe this report should be published promptly, I provide some comments below.

**RESPONSE:** We would like to thank the reviewer for the insightful reviews, particularly for the positive comments regarding the utility of the reference data set generated by this study.

1. Using consensus calling across multiple callers for a type of SV reduced false positive rate at the expense of sensitivity. This strategy will certainly provide a very good call set, but one wonders what are we missing. Can you provide an estimate of the false negative rate of the consensus call set?

**RESPONSE:** Thank you very much for your helpful comment and suggestion. We agree that using a consensus calling approach reduces the false positive rate but at the expense of sensitivity. To date, there is no universal method that can provide accurate detection for the full spectrum of SV types and sizes, especially for cancer genomes due to complex rearrangement in chromosome structure as well as tumor cell heterogeneity. From our study, we found an integrative approach combining SVs from multiple complementary technologies and multiple software tools provided the most comprehensive view of the structural variants in the reference cancer line. Yes, we concede that the stringent protocol which requires at least two technologies from two software tools to agree on the same variant called for our high confidence call set, which can cause increased false negative calls. We acknowledged this limitation on our study and added the following sentence in the discussion section:

“*Our high confident call set requires at least two technologies to agree on the same structural variant based on SV types and SV sizes, while the stringent protocol works better for establishing a high-quality call set, but it is at the expense for detection sensitivity. We acknowledge that there is a need for continuous improvement of the SV call set for the HCC1395 cancer genome as new technologies and more accurate SV calling algorithms and pipelines become available*”.

The lack of truth set for the cancer reference cell line prevent us from calculating a false negative rate. However, we performed the assessment of relative sensitivity for SV call across platforms from our initial consensus call set by computing the proportion of all calls called by a reference platform recoverable using another technology. We provide detailed benchmarking result in the following section.

**Suppl. Fig. 6. Relative Sensitivity for SV Calls Across Platforms.** **(a)** Comparison of SVs called by different platform including Nanopore, PacBio, Illumina short-read, Bionano, 10X Chromium linked-read, and HiC. Upset plot expanding on data similarities highlighted in **Figure 2a**. Intersection size is split into SV-type including multiple breakpoints SV events (BND), deletions (DEL), duplications (DUP), insertions (INS), inversions (INV), and inter-chromosomal translocations (TRA). Violin plots indicate the distribution of SV-sizes, in base-pairs (bp), within the vertically aligned with overlap-sets; horizontal lines within the violin plots indicate the 1^st^ quantile, median, and 3^rd^ quantile. A horizontal line is included in the violin plots to indicate 100kb size. **(b)** Relative sensitivity was calculated for all SV calls. **(c)** Relative sensitivity was calculated for all SV calls and stratified by size, between x and x*10 base pairs (x-axis) where x is the vertically aligned value on the x-axis. Relative sensitivity (x-axis in **b,** y-axis in **c**) is the proportion of all SV calls include SVs from initial call set defined in **Supplementary Table 3** and Bionano call set from **Supplementary Table 14** called by a reference platform (color) recoverable using another technology (y-axis in **b**, sub-panels in **c**) across different platforms.

Overall relative sensitivity indicates high concordance between Nanopore and Pacbio SV calls (>75% PacBio calls is recoverable from Nanopore calls). Illumina SV calls showed moderate concordance with 10X and Bionano while 10X and Bionano showed limited power to recapitulate Illumina SV calls. We found that Illumina short-read technology predicted fewer than 25% of Pacbio and Nanopore SV calls smaller than 1kbp and predicted over 75% of Pacbio and Nanopore SV calls between 5kbp and 500kpb. Conversely, Pacbio and Nanopore predicted close to 50% of Illumina SV calls regardless of size. Illumina, Pacbio and Nanopore provided the most redundant calls for small and moderate size SV. Bionano and 10x calls appeared to be more similar; up to 50% of Bionano calls could be predicted by 10x between 50kbp and 500kbp while over 45% of 10x SV calls larger than 1Mbp could be predicted by Bionano. 10x predictive power appeared to peak for SV calls larger than 100kbp yet 10x could not predict most Bionano SV calls (relative sensitivity <30%) regardless of size. Interestingly, Bionano SV calls larger than 500kbp were the hardest to predict and therefore the most unique from any platform (relative sensitivity < 50%). Yet, the predictability of similarly sized 10x SV calls increased for larger SV calls as Bionano predictability decreases.

Taken together, the above analysis suggests that while there is low concordance across different technologies and software tools, there was consistency for specific SV types and size ranges for certain platforms. While using multiple tools and consensus approaches helped to reduce the false positive rates for SV detection, we should also consider the unique strength of each technology and apply complementary technologies to increase the SV detection sensitivity and spectrum in cancer genomes.

2.    The results about the impact of mixtures between normal and tumor cell lines is somehow embedded in the discussion section, while I think this is a very important results and merits its own section and figures rather than in supplemental materials. This analysis is very relevant to those trying to design assays to sequence tumor tissue with variable rates of purity. I would suggest a figure that plots the sensitivity across different rates of mixtures, with lines representing different types of variants and at various depths of coverages (may be separate panels for different depths) to make very clear the impact of mixture levels vs depth of coverage, quite useful for practical purposes. This could be a figure in the main body of the paper rather than just supplemental tables instead of supplemental tables. Additional supplemental figures could also provide similar analysis for different platforms. I think this information would be one of the major learnings of this work.

**RESPONSE:** We thank the reviewer for the great suggestion and comments. We fully agree these are very important results and merit their own section. We have added a section “Tumor Purity Impact for SV Detection Sensitivity” and added Figure 5 to show the results. We plotted the figure as the reviewer suggested, using lines representing different types of SVs at various percentages of tumor purity. We use the panels for 10x, 30x, 50x, 100x, 200x and 300x to show the impact of mixture levels vs. sequencing depth.

**Figure 5: Effect of tumor purity and sequencing depth for SV detection. (a)** Compare small SVs (50bp -30kb size range) detection sensitivities in different tumor purities in 5%, 10%, 20%, 50%, 75% and 100% tumor purity with 100x sequencing coverage. (**b**) Compare Large SVs (>30kb) detection sensitivities in different tumor purities in 5%, 10%, 20%, 50%, 75% and 100% tumor purity at 100x sequencing coverage. The blue horizontal bars on the left show the total number of SVs in the specific tumor purity, pink bar with black dots denote number of SVs called in each tumor purity data. The top black vertical bars display the total concordance calls among the different tumor purities. (**c**) line charts display SV detection sensitivity among different sequencing depth (10x, 30x, 50x, 100x, 200x, 300x) and different tumor purities (5%, 10%, 20%, 50%, 100%). The reference call set was built with consensus methodology and used the SVs from our consensus call set. The recalled SVs were separated into TRA (Translocation), DEL (Deletion), DUP (Duplication) and INV (Inversion).

For the tumor mixture experiment, we prepared Illumina WGS libraries only, therefore, only Illumina short-read data sets are available and no other platform data are available.

3.    Figure 2's legend appears to have some errors. References to the panels d to g appear to be mislabeled.

**RESPONSE:** We thank the reviewer for catching up the mislabeling. We have now corrected Figure 2 legend for panels d – g.

4.    Can you describe in more detail how you benchmarked the comparisons between the consensus reference call set and a given technology call set. Benchmarking a reference set of SV calls to a second call set is not a trivial problem and the methods used to perform this function need to be described and validated.

**RESPONSE:** We thank the reviewer for excellent suggestion. Yes, we have performed the suggested analyses and documented the comparison between the consensus reference call set and a given technology call set. We also provided a paragraph as showed below, we thought the results are important to show as a separate section in the Result. We thus would like to leave the final decision to the Editor or the reviewers’ preference if it should be included in the main manuscript.

Briefly, we used our initial consensus call set as reference call set including PacBio, Nanopore, Illumina short-reads, Bionano, 10X Chromium linked-reads, and HiC called SVs as defined in **Supplementary Table 3** and Bionano call set in **Supplementary Table 14.** We measured the inter-platform relative sensitivity (**Suppl. Fig. 6b**) by computing the proportion of all calls called by a reference platform that was reproduced by another technology. We selected the initial consensus call set rather than the high-confidence call set as reference call set considering the following: platform unique SVs which were not detected by other platform were filtered out from the high-confidence call set. This could largely increase cross-platform concordance rates. Using the initial consensus call set as reference call set was closely matched to the default call sets from individual tools/platforms, therefore, can better reveal each platform’s unique strength and weakness for the SV detection.

**Extra figure for Reviewer only**: **Relative Sensitivity of “predictor’ to recover ‘reference’ SVs.** All panels include all SV calls across platforms including Nanopore, PacBio, Illumina short-read, Bionano, 10X Chromium linked-read, and HiC. The initial consensus call set defined in **Supplementary Table 3** and Bionano call set from **Supplementary Table 14** was used as all SV call set. **(a)** Relative sensitivity (y-axis in a) is the proportion of all SV calls called by a reference platform (color) recoverable using another technology (sub-panels in a and b) across different platforms. **(b)** Ribbon plot expansion on relative sensitivity to show the numerator (smallest shaded y-coordinate; number of reference SVs (color) found by the predictor platform (subpanel)) and the denominator (largest shaded y-coordinate; number of reference SVs (color)); the numerator at each size interval is the smallest vertically aligned y-coordinate within the shaded region while the denominator is the largest vertically aligned y-coordinate.

The overall relative sensitivity (**Suppl. Fig. 6b**) indicated high concordance between Nanopore and PacBio SV calls (>75% PacBio calls are reproducible from Nanopore calls). Illumina SV calls showed moderate concordance with 10X and Bionano while 10X and Bionano demonstrated limited power to recapitulate Illumina SV calls. HiC SV calls in our initial call set were redundant (>80% for Illumina and 10X) with all platforms, which was partially due to HiC Selva unique calls being filtered as the initial consensus call set required at least 2 software tools or 2 replicates making the same call. Stratifying relative sensitivity by SV size (**Suppl. Fig. 6c**), we found that Illumina short-read technology predicted fewer than 25% of PacBio and Nanopore SV calls smaller than 1kbp and predicted over 75% of PacBio and Nanopore SV calls between 5kbp and 500kpb. Conversely, PacBio and Nanopore predicted close to 50% of Illumina SV calls regardless of size. Illumina, PacBio and Nanopore provided the most reproducible calls for small and moderate size SV. Bionano and 10x calls appeared to be more similar; up to 50% of Bionano calls could be predicted by 10x between 50kbp and 500kbp while over 45% of 10x SV calls larger than 1Mbp could be predicted by Bionano. 10x predictive power appeared to peak for SV calls larger than 100kbp yet 10x cannot predict most Bionano SV calls (relative sensitivity <30%) regardless of size. Interestingly, Bionano SV calls larger than 500kbp were the hardest to predict and therefore the most unique from any platform (relative sensitivity < 50%). Yet, the predictability of similarly sized 10x SV calls increased for larger SV calls as Bionano predictability decreased. While we note that Nanopore and PacBio discovered fewer than 25% of small Illumina SV calls, this is less related to the orthogonality of the platforms than the large number of Illumina platform unique SV calls and the relatively small number of those SV calls reproduced by PacBio or Nanopore.

**Second round of review**

**Reviewer 1**

Thanks for the author's responses, and most of my concerns have been addressed. However, I still have the following questions regarding to the merge process and validation.

1. For your initial callset integration, I found you merged somatic short deletions from 4 files separately but it is unclear how the insertions are merged. Moreover, what if different criterion is applied to split VCF file, e.g., 50bp to 300bp, 300bp to 1000bp, etc., will that affect the clustering analysis or even the final high confident set? I think further clarification would be one of the major contributions of this work to the community.

2. I am curious about the technology specific SVs, are they induced from inaccurate merge or other reasons? As far as I know, SURVIVOR merges SVs based on size, breakpoint difference and type, it is likely that duplications are reported as insertions, which won't be merged accordingly. And I noticed that your high confident SV set contains duplications and insertions.

3. The author mentions complex breakpoint events, which are prevalent in tumor and difficult to merge between sequencing technologies. In your example (chr3:79945732-85636036), it is likely that every BND is merged independently and the whole internal structures of the event is not considered. Moreover, this complex event spans a wide range of genome, and its breakpoints might originated from subclonal tumor cell populations as mentioned in the response. What about complex event like inverted duplication, deletion associated with inversion, etc., that have been reported by 1000 Genomes Project in their 2015 Nature publication, is your merge process affected such events that could be covered by a read?

4. For the validation part, I am curious why the validation rate for TRA is only 25%?

**Reviewer 2**

The authors’ rebuttal and revised manuscript has addressed my original concerns and questions. The new sections/figures are informative and complement the original manuscript. I believe the current manuscript is ready to be published. However, I would strongly suggest that the figures provided for reviewers only be included in the supplementary materials. I found these useful in addressing concerns that readers may have and additive to current figures. I also repeat my suggestion (item 4) to include details of the benchmarking approach in the published paper, whether in the main text or in the supplemental materials.

**Authors Response**

**Point-by-point responses to the reviewers’ comments:**

**Reviewer 1:**

Thanks for the author's responses, and most of my concerns have been addressed. However, I still have the following questions regarding to the merge process and validation.

*RESPONSE: We sincerely thank the reviewer for spending time to review our manuscript and providing these insightful suggestions and helpful comments regarding the SV merge process and validation*.

1. For your initial callset integration, I found you merged somatic short deletions from 4 files separately but it is unclear how the insertions are merged. Moreover, what if different criterion is applied to split VCF file, e.g., 50bp to 300bp, 300bp to 1000bp, etc., will that affect the clustering analysis or even the final high confident set? I think further clarification would be one of the major contributions of this work to the community.

*RESPONSE: Insertions, deletions, duplications and inversion events are merged based on the window sizes (50 to100bp, 101to 500bp, 501 to 1000bp, 1001 to 30kb) for small SVs (50bp - 30kb sizes) using the same criteria. The translocation events and large SVs (over 30kb) were processed separately using a 10kb window size for merging. The previous methods section detailing the referring window bin method only for somatic short deletions was inaccurate. The method section is now updated to provide more detail for the SV merge process as follows:*

*Multi-platforms SV Integration The somatic SV events such as insertions, deletions, intra-chromosomal inversions, inter-chromosomal translocations, and complex breakpoint events were called in a tool-specific manner in the above sections described in platform/tool specific SV analysis. If somatic calling mode was not supported by a specific software, Survivor [25] v1.0.3 SVs was used to get SVs in tumor sample and not in the normal sample for each pair of the sample. Split VCF files The SVs in each tool specific VCF file were split based on the 5 window sizes (50 to100bp , 101to 500bp, 501 to 1000bp, 1001 to 30,000bp, 30,000bp and above). Translocation events (TRA) were extracted from the split VCF file to save into a separate file for each window bin group. The Survivor filter function was used to split VCF files as following: SURVIVOR filter $input.vcf NA $min_size $max_size 0 -1 $window_bin/$output.vcf && cat $window_bin/$output.vcf | grep -w -v "SVTYPE=TRA" | vcf-sort >$window_bin/$output.noTRA.vcf && cat $window_bin/$output.vcf | grep -w "SVTYPE=TRA" | vcf-sort >$window_bin/$output.only.TRA.vcf*

*Somatic Large SV*

*To get the tumor only (somatic) SVs, the large SV (SV >30kb) VCF files for each pair were merged using Survivor. The SVs were merged if they were <10kb between the break points. SVs which were only present in the tumor samples and not in the normal sample were taken as somatic SVs for each pair of the sample. The command used for merging the VCF files for SV integration by window sizes (10,000bps) was: Command: SURVIVOR merge $input.vcf.list 10000 1 0 0 0 10000 $output.vcf*

*Somatic Small SV: To get tumor specific small deletions, insertions, duplications and inversions, the VCF file per sample was split into 4 files based on the length of the SV size: 50bp to 100bp, 101bp to 500bp, 501bp to 1KB and SV (size) greater than 1kb but smaller than 30kb. Each of the pairs of samples were then merged using Survivor. Their minimum SV length was used as the maximum distance between the break point. The resulting tumor specific SVs were then merged from all the four files as tumor specific SVs for each pair. The command used for merging the VCF files for SV integration by window sizes (50, 100, 500, 1000) was:*

*SURVIVOR merge $input.vcf.list $window_size 1 0 0 0 $window_size $output.vcf Merging Somatic Call Sets: Large SVs (SV > 30Kb or Translocations) from the Tumor only filter calls from all samples from all the tools (11 LongRanger, 11 GrocSV[59], 11 TNScope,11 Novobreak, 11 Delly, 1 Sniffles and 1 PBSV for PacBio data, 1 Sniffles and 1 NanoSV for ONT data, and 1 Dovetail HiC ) were merged using the same command for large SV integration from Survivor:*

*Command: SURVIVOR merge $input.vcf.list 10000 1 0 0 0 10000 $output.vcf Small SVs (SV < 30Kb and No Translocations) from tumor only calls from all samples and all tools (11 LongRanger for short deletions, 11 TNScope, 11 Novobreak, 11 Delly, 1 Sniffles and 1 PBSV for PacBio data, 1 Sniffles and 1 NanoSV for ONT data) VCF files were merged with the same command for small SV integration from Survivor as follows:*

*SURVIVOR merge $input.vcf.list $window_size 1 0 0 0 $window_size $output.vcf In addition, SVs on ALT contigs or 10X Genomics’ blacklist regions were removed. For the high confidence SV call set, we selected SVs that were represented in calls from at least 2 technologies and window size of same bin for merging the SVs of the same type together.*

*The SVs are clustered by the same criteria as well. That is, insertions and deletions (and other types too) are clustered according to this following scheme:*

*50bp to 100bp SV => 50bps clustering window size*

*100bp to 500b SV => 100bps clustering window size*

*500bp to 1000 SV => 500bps clustering window size*

*1001bp to 30000bp SV => 1kb clustering window size*

*30kb above SV => 10kb clustering window size*

*translocations => 10 kb clustering window*

*As to the effect of using different bins, we agree with the reviewer that the subsequent clustering analysis would be affected. Those variants whose size is near a bin boundary may not be clustered optimally, because the equivalent variants may be precategorized into different bins. For example, two insertions, 295 bp and 301 bp, would be clustered/merged if the bin window is 100bp to 500 bp, but they would not be merged had the bin windows been 100bp to 300bp and 300bp to 500bp.*

2. I am curious about the technology specific SVs, are they induced from inaccurate merge or other reasons? As far as I know, SURVIVOR merges SVs based on size, breakpoint difference and type, it is likely that duplications are reported as insertions, which won't be merged accordingly. And I noticed that your high confident SV set contains duplications and insertions.

*RESPONSE: We thank the reviewer for pointing out the challenges in the SV merge process and limitations of Survivor merge. The technology specific SVs in the initial call set were mainly due to NGS technology and software algorithm difference, but it could also be the result of an inaccurate merge.*

*We observed that different software tools might call the SV overlap the same region as different events or ignore a specific type of event. For example, Longranger only calls small deletions for small SVs between 50bps - 30kb. While it calls deletions, inversions, and duplication and break ends events for large SVs (>30kb), it does not report insertion and translocation events for large SVs. On the other hand, PBSV and Sniffles both report deletions, insertions, duplications, inversion and translocations for the PacBio data set. Comparing SVs from Illumina to other platforms, we noticed Illumina detected a higher proportion of platform specific translocation events and deletions in the range of 500bps – 1kb in the initial call set. However, Nanopore and PacBio detected high proportions of platform specific small insertions between 50bps – 1kb, Bionano reported a high proportion of platform specific SVs as duplications between 100kb to several megabases.*

*To avoid spliting the similar size overlap SVs into different SVs, we applied a clustering approach to further merge SVs of the same type if the SVs had a reciprocal overlap >50%. By using this clustering approach, we removed 79 additional SVs that were missed by the Survivor merge approach in high confidence consensus call set. The 79 SVs includes 1 deletion, 1 duplication, 1 Insertion, 27 reciprocal Inversions and 49 reciprocal translocation events. We performed manual curation to resolve the conflicts for some SVs with types of differences.*

3. The author mentions complex breakpoint events, which are prevalent in tumor and difficult to merge between sequencing technologies. In your example (chr3:79945732-85636036), it is likely that every BND is merged independently and the whole internal structures of the event is not considered. Moreover, this complex event spans a wide range of genome, and its breakpoints might originated from subclonal tumor cell populations as mentioned in the response. What about complex event like inverted duplication, deletion associated with inversion, etc., that have been reported by 1000 Genomes Project in their 2015 Nature publication, is your merge process affected such events that could be covered by a read?

*RESPONSE: We thank the reviewer for the insightful comment regarding challenges in merging complex breakpoint events between sequencing technologies and the limitations of the Survivor algorithm for handling SVs originating from subcolonal tumor cell populations. For complex breakpoint events, Survivor might not merge them together due to SV type and size differences called by different software tools from different technologies. Those complex events, such as inverted duplications, deletions associated with inversions, could be reported as separate BNDs or multiple overlap SVs. We added this point into the Discussion to acknowledge the limitation with our current study:*

*“Due to the current technology and software algorithm limitations, some of the complex breakpoint events were not consistently called across technologies and need to be further characterized (Additional file 10: Fig. S9, Additional file 16: Table S15). We acknowledge that there is a need for continuous improvement of the SV call set for the HCC1395 cancer genome as new technologies and more accurate SV calling algorithms and pipelines become available.”*

4. For the validation part, I am curious why the validation rate for TRA is only 25%?

*RESPONSE: The validation rates for the DEL, DUP, INV, and TRA were 93%, 75%, 54% and 25%, respectively based on PCR validation results for a total of 110 SV events from different size ranges from the initial call set. Among the 18 translocation events to not validated by PCR, 14 were in highly repetitive regions. As we previously clarified, the incapable detection of one SV by a chosen validation method does NOT necessarily mean a true false positive call. For translocation event confirmation, fluorescent in situ hybridization (FISH) remains the gold standard for assessing recurrent translocations and has much higher positivity compared to PCR validation as reported by Espinet B, et al. Leuk Res. 2018 and others. The additional challenge arises because the breakpoint regions might be present at the repetitive region or near centromere or other SV events. In both these conditions, PCR amplifications are difficult to obtain. Based on Bionano results, 39% of the translocation events in our consensus call set were validated by Bionano. As demonstrated in our study as well as by other studies such as Dixon, J.R. et al. Nat Genet. 2018, to date, there is still no universal method that can provide an accurate detection for the spectrum of SV type and size.*

**Reviewer 2**

The authors’ rebuttal and revised manuscript has addressed my original concerns and questions. The new sections/figures are informative and complement the original manuscript. I believe the current manuscript is ready to be published. However, I would strongly suggest that the figures provided for reviewers only be included in the supplementary materials. I found these useful in addressing concerns that readers may have and additive to current figures. I also repeat my suggestion (item 4) to include details of the benchmarking approach in the published paper, whether in the main text or in the supplemental materials.

*RESPONSE: We sincerely thank the reviewer for the insightful comment and suggestions to make our manuscript better. Yes, we have included in the manuscript the explanations which were in the response to reviewers in previous revision. Specifically, we added a section titled “Assessment of Relative Sensitivity for SV Calls Across Platforms” to evaluate the performance of different platforms. All the figures are included in Additional file 10, Fig. S6a, b, c, d. In addition, we added a point of discussion on the technical limitation of each technology and the computational challenges associated with the complex SV detection. We add figures and tables to describe the complex BNDs need to be further characterized in Additional file 10: Fig. S9, Additional file 16: Table S15.*
